# Aberrant expression of SLAMF6 constitutes a targetable immune escape mechanism in acute myeloid leukemia

**DOI:** 10.1038/s43018-025-01054-6

**Published:** 2025-10-03

**Authors:** Carl Sandén, Niklas Landberg, Pablo Peña-Martínez, Hanna Thorsson, Shruti Daga, Noelia Puente-Moncada, Maria Rodriguez-Zabala, Sofia von Palffy, Marianne Rissler, Vladimir Lazarevic, Gunnar Juliusson, Mats Ohlin, Axel Hyrenius-Wittsten, Christina Orsmark-Pietras, Henrik Lilljebjörn, Helena Ågerstam, Thoas Fioretos

**Affiliations:** 1 Division of Clinical Genetics, Department of Laboratory Medicine, Lund University, Lund, Sweden; 2 Department of Hematology, Oncology and Radiation Physics, Skåne University Hospital, Lund, Sweden; 3 Department of Immunotechnology, Lund University, Lund, Sweden; 4 SciLifeLab Drug Discovery and Development Platform, Lund University, Lund, Sweden; 5 Department of Clinical Genetics, Pathology, and Molecular Diagnostics, Skåne University Hospital, Region Skåne, Lund, Sweden

**Keywords:** Acute myeloid leukaemia, Cancer, Antibody therapy, Tumour immunology

## Abstract

Immunotherapy has shown limited success in acute myeloid leukemia (AML), indicating an incomplete understanding of the underlying immunoregulatory mechanisms. Here we identify an immune evasion mechanism present in 60% of AML cases, wherein primitive AML cells aberrantly express the lymphoid surface protein SLAMF6 (signaling lymphocyte activation molecule family member 6). Knockout of *SLAMF6* in AML cells enables T cell activation and highly efficient killing of leukemia cells in coculture systems, demonstrating that SLAMF6 protects AML cells from recognition and elimination by the immune system in a mode analogous to the programmed cell death protein–ligand (PDL1/PD1) axis. Targeting SLAMF6 with an antibody against the SLAMF6 dimerization site inhibits the SLAMF6–SLAMF6 interaction and induces T cell activation and killing of AML cells both in vitro and in humanized in vivo models. In conclusion, we show that aberrant expression of SLAMF6 is a common and targetable immune escape mechanism that could pave the way for immunotherapy in AML.

## Abstract (web summary)

Sandén et al. report that the ligand signaling lymphocyte activation molecule family member 6 protects acute myeloid leukemia cancer cells from T cell-mediated killing, leading to immune escape, and show that this can be targeted by a blocking antibody.

## Main

Acute myeloid leukemia (AML) is a genetically heterogenous disease initiated and propagated by leukemia stem cells (LSCs). These cells are refractory to current treatments with chemotherapy and hypomethylating agents and provide a source for disease relapse following current treatment protocols^1,2^. The prognosis in AML is poor, with a 5-year survival rate of 30%, emphasizing the need for novel therapeutic approaches^3,4^. The recent advances in cancer immunotherapy have generated remarkable results in many tumor types but only modest benefits in AML. There is, therefore, a great need for improved understanding of the complex interplay between tumor cells and the immune system in AML.

AML is associated with an impaired anticancer T cell response but the underlying mechanisms are still largely unknown. In general, persons with AML have T cell numbers comparable to those in normal bone marrow (NBM) and these include tumor-reactive T cells^5–8^. Moreover, common AML neoantigens such as mutated *NPM1* and *IDH1* have been shown to induce T cell activity^9,10^. However, the T cell landscape is commonly affected in AML and cases can be divided into two groups of similar size on the basis of immune infiltration characteristics. The immune ‘infiltration subgroup’ displays an interferon-γ gene signature and high expression of T cell cytotoxic molecules such as perforin and granzyme B, whereas the ‘immune depletion’ subgroup is characterized by expression of the memory marker CD45^RO^ and the T cell exhaustion marker programmed cell death protein 1 (PD1)^11^. Concordantly, the frequency of T cells expressing the exhaustion markers PD1, lymphocyte activation gene 3 (LAG3) and T cell immunoglobulin and mucin domain-containing protein 3 (TIM3) has been shown to be increased in a subset of AML cases at diagnosis and further increased at relapse^8,12^. Similarly, the early memory CD8^+^ T cell population that contributes to therapy response displays two distinct trajectories with activation markers dominating in one subset of persons and senescence markers in the other^13^. On the basis of surface marker expression and cytokine profiling, cells with phenotypic profiles of exhaustion and senescence represent two distinct T cell populations in AML that are both enriched compared to NBM^14^. In addition to these characteristics, persons with AML have been shown to have reduced frequencies of naive T cells and increased levels of regulatory T cells with enhanced suppressive functions^8,14,15^. The T cell landscape seems to be at least partly related to genetic aberrations, as patients with mutations in *TP53* or myelodysplasia-related genes such as *RUNX1* display lower infiltration of cytotoxic T cells and more pronounced immune effector dysfunction^16,17^.

AML cells have in some cases been shown to directly induce T cell suppression through expression of ligands for coinhibitory receptors. PD1 ligand 1 (PDL1) is expressed in around 10% of cases and is most frequently upregulated in persons with *TP53* mutations^8,18–21^. The cytotoxic T lymphocyte-associated protein 4 (CTLA4) ligand CD86 is also expressed in a subset of cases, with expression most common in CD34^−^ and monocytic leukemias^20,22–24^. The T cell composition in AML has been correlated with treatment outcome. High levels of T cells in the leukemic bone marrow have been associated with better survival^25,26^. However, extensive T cell infiltration has also been coupled to poorer response to standard chemotherapy, especially for cases with unfavorable genetic aberrations such as *TP53* mutations^11^. Low levels of regulatory T cells (Tregs) and high degrees of T cell exhaustion and senescence have also been associated with poor outcome^13,16,27–29^. Contrarily, patients with immune infiltration have been shown to be more responsive to T cell-based therapies such as PD1 inhibitors and bispecific T cell-engaging antibodies^11,27^. Despite the T cell dysregulation in AML, current immunotherapies targeting PD1, PDL1 and CTLA4 have proven insufficient to restore T cell function and have produced very limited clinical benefits^30–33^. The modest responses suggest that LSCs may harbor other critical immune escape mechanisms. Hence, identifying and targeting these pathways constitutes the major challenge to successful immunotherapy in AML.

In this study, we identify an immune escape mechanism in AML that has not previously been observed in any type of cancer. We show that 60% of AML cases across all major genetic subgroups aberrantly express the cell surface protein signaling lymphocyte activation molecule family member 6 (SLAMF6). SLAMF6 is normally involved in the regulation of lymphocyte activation, where it forms a homodimer between SLAMF6 molecules on two interacting T cells to moderate their activity^34–37^. We demonstrate that AML cells mimic this mechanism to suppress the antileukemic T cell response. Knocking out *SLAMF6* on AML cells with CRISPR–Cas9 induces activation of interacting T cells and renders the AML cells susceptible to T cell-mediated killing. Consistent with the immunosuppressive role, we show that persons with AML expressing SLAMF6 display altered T cell compositions with increased frequencies of naive T cells compared with other AML cases. To target the SLAMF6 immune checkpoint, we developed a fully human SLAMF6 antibody that binds the homodimerization interface with high affinity and disrupts the SLAMF6–SLAMF6 interaction. Treatment with the SLAMF6 antibody potently induces T cell-mediated killing of AML cells both in vitro and in humanized in vivo models. In conclusion, we identify SLAMF6 upregulation as a common immune escape mechanism in AML and show that SLAMF6 antibodies targeting this interaction can unleash a strong anticancer T cell response.

## Results

### SLAMF6 is aberrantly expressed on primitive AML cells

**Fig. 1:**
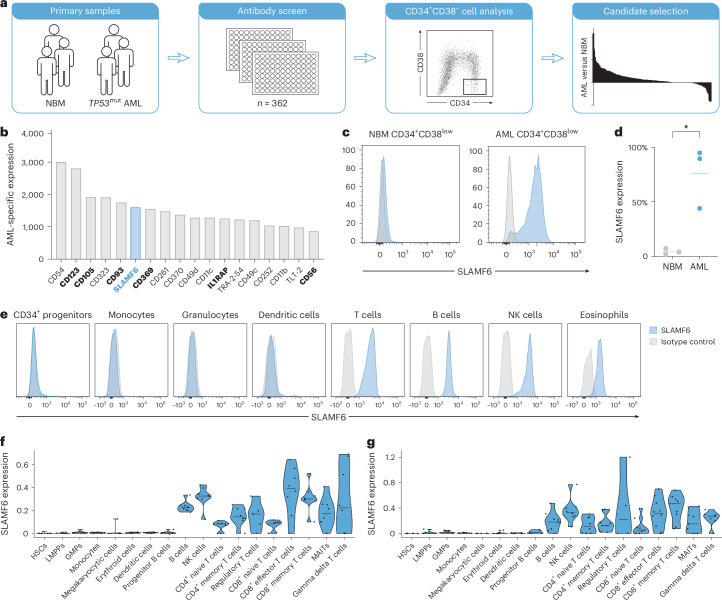
SLAMF6 is expressed on AML stem cells. **a**, Schematic overview of the antibody screen to identify novel markers differentially expressed on CD34^+^CD38^low^ cells from NBM and from participants with AML with *TP53* mutations by flow cytometry. **b**, Most highly expressed cell surface markers on CD34^+^CD38^low^ cells in AML (*n* = 3 participants) compared to NBM (*n* = 3 donors) samples. Previously identified AML markers are shown in bold. **c**, Expression of SLAMF6 on CD34^+^CD38^low^ cells from representative samples from NBM and a participant with AML. **d**, SLAMF6 expression on the three AML cases and three NBM samples included in the antibody screen (two-sided *t*-test, *P* = 0.011). **e**, Expression of SLAMF6 on normal hematopoietic cell populations in peripheral blood from healthy donors (one representative donor of four). **f**, Expression of SLAMF6 in hematopoietic cell types based on scRNA-seq of mononuclear cells from five NBM donors and CD34^+^ cells from three donors. In the violin plot, dots represent individual donors, bars indicate median values and blue shading indicates density. **g**, SLAMF6 expression in NBM populations from an scRNA-seq dataset with ten healthy donors^29^. In the violin plot, dots represent individual donors, bars indicate median values and blue shading indicates density. Source Data

**Extended Data Fig. 1:**
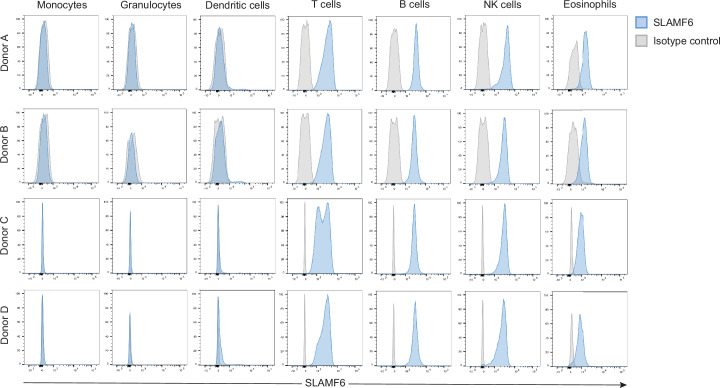
SLAMF6 expression on normal hematopoietic cells. Expression of SLAMF6 on normal hematopoietic cell populations in peripheral blood from four healthy donors.

**Extended Data Fig. 2:**
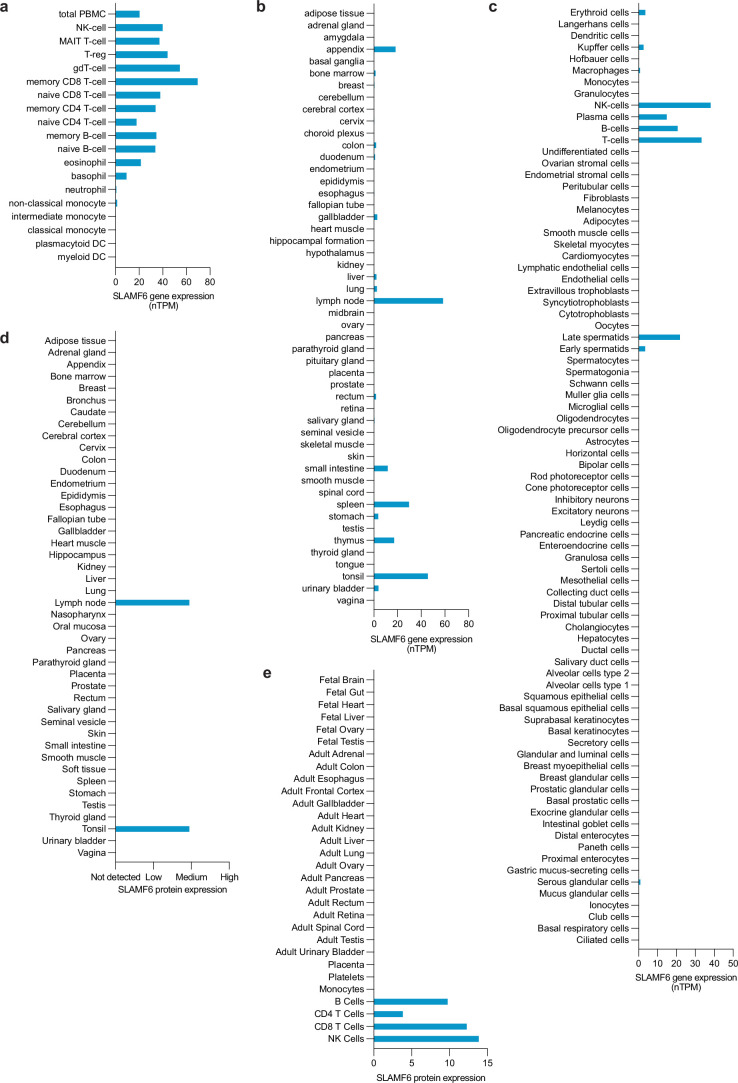
SLAMF6 gene and protein expression in normal cells. (**a**) SLAMF6 expression in immune cell populations based on the”Immune Cells” RNA sequencing dataset from the Human Protein Atlas. (**b**) Gene expression of SLAMF6 in major tissues based on the Human Protein Atlas RNA sequencing dataset. (**c**) SLAMF6 gene expression in major cell types in the”Single Cell Type” whole-body scRNAseq dataset from the Human Protein Atlas. (**d**) SLAMF6 protein expression in major tissues based on immunohistochemistry in the”Human Protein Atlas” dataset. (**e**) Protein expression of SLAMF6 in major tissues and cell types based on mass spectrometry in the”Human Proteome Map” dataset. Source Data

To identify cell surface proteins specifically expressed on primitive AML cells, we used an arrayed antibody screening system to determine the expression of 362 different cell surface markers within the primitive CD3^−^CD19^−^CD34^+^CD38^low^ cell population in *TP53*-mutated AML bone marrow samples and healthy NBM controls (Fig. 1a). Candidate cell surface markers were ranked on the basis of a combined metric of mean fluorescence intensity (MFI) and fraction of positive cells in the primitive compartments of AML and NBM (Fig. 1b). The top candidates included known AML markers CD123 (IL3RA)^38^, CD105 (Endoglin)^39^, CD93 (ref. ^40^), CD369 (CLEC7A)^41^, IL1RAP^42,43^ and CD56 (NCAM1)^44^, as well as multiple novel candidate markers not previously described in AML. In validation experiments with the top candidates, SLAMF6 was confirmed to be highly expressed on primitive CD34^+^CD38^low^ AML cells but not on corresponding healthy hematopoietic stem and progenitor cells (HSPCs) from NBM (Fig. 1c, d). Further characterization of the expression on normal hematopoietic cells confirmed that SLAMF6 was also absent on normal CD34^+^ progenitor cells, dendritic cells, monocytes and granulocytes and only present on lymphocytes (T, B and natural killer (NK) cells) and eosinophils (Fig. 1e and Extended Data Fig. 1). This expression pattern was confirmed by *SLAMF6* gene expression profiling in an in-house single-cell RNA sequencing (scRNA-seq) dataset with CD34^+^ and mononuclear cells from five NBM samples^45^, as well as in an analogous public dataset^29^ and in the ‘immune cells’ RNA-seq dataset from the Human Protein Atlas (Fig. 1f,g and Extended Data Fig. 2a). Analysis of additional Human Protein Atlas and Human Proteome datasets showed that SLAMF6 is not expressed in other human tissues according to RNA-seq, scRNA-seq and protein expression by immunohistochemistry and mass spectrometry (MS) (Extended Data Fig. 2b–e). We conclude that SLAMF6 is aberrantly expressed on the surface of primitive AML cells but not on healthy HSPCs or nonhematopoietic cells.

### SLAMF6 is expressed across AML subtypes

**Fig. 2:**
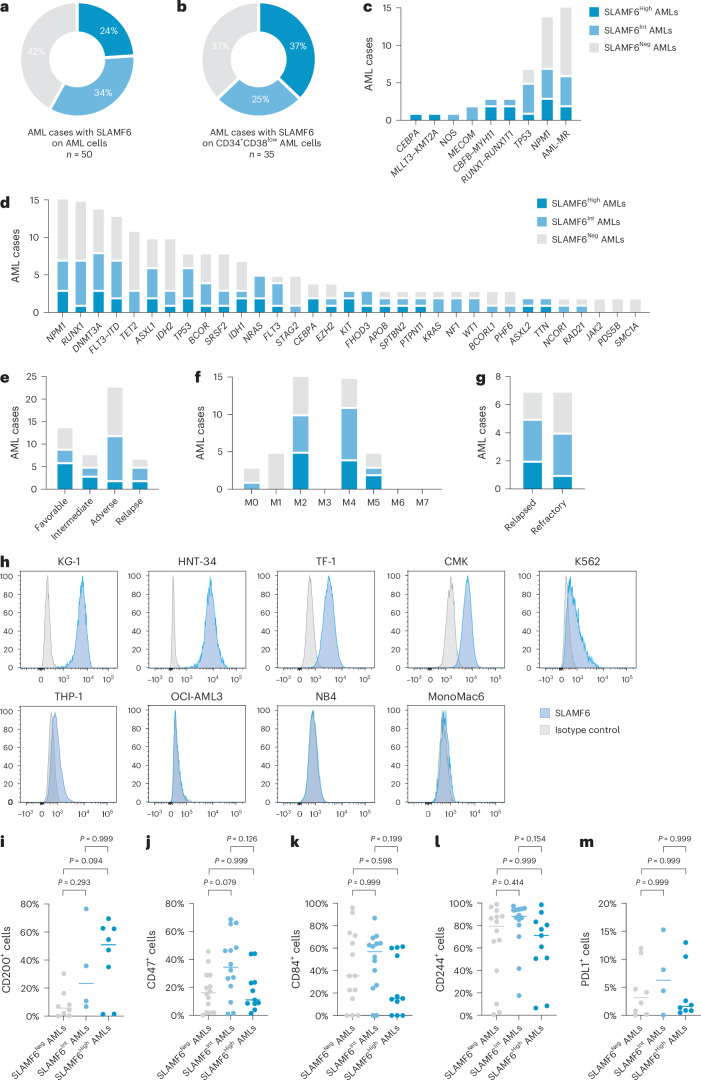
SLAMF6 is expressed in a wide spectrum of AML cases. **a**, Percentage of AML samples expressing SLAMF6 on the CD33^+^ blast cell population (*n* = 50 cases). **b**, Percentage of the CD34^+^ AML samples expressing SLAMF6 on the CD34^+^CD38^low^ cell population (*n* = 35 cases). Frequency of AML cases expressing SLAMF6, stratified by disease subtype as defined by the ICC 2022 classification (**c**), recurrent mutations (**d**), ELN 2022 risk group classification (**e**) and French–American–British classification (**f**). **g**, SLAMF6 expression on cases of relapsed/refractory AML. Dark blue, high SLAMF6 expression; light blue, intermediate SLAMF6 expression; gray, no SLAMF6 expression. **h**, SLAMF6 expression on myeloid leukemia cell lines by flow cytometry. Blue, staining with SLAMF6 antibody; gray, staining with isotype-matched control antibody. Correlation between the expression of SLAMF6 and that of CD200 (**i**), CD47 (**j**), CD84 (**k**), CD244 (**l**) and PDL1 (**m**) on primary AML samples, based on flow cytometry (Kruskal–Wallis test with Dunn’s post hoc test; *n* = 20 cases for CD200 and PDL1 and *n* = 39 cases for CD47, CD84 and CD244). Bars indicate median values. *CEBPA*, AML with in-frame bZIP *CEBPA* mutations; *MECOM*, AML with other *MECOM* rearrangements; NOS, AML not otherwise specified. Source Data

**Extended Data Fig. 3:**
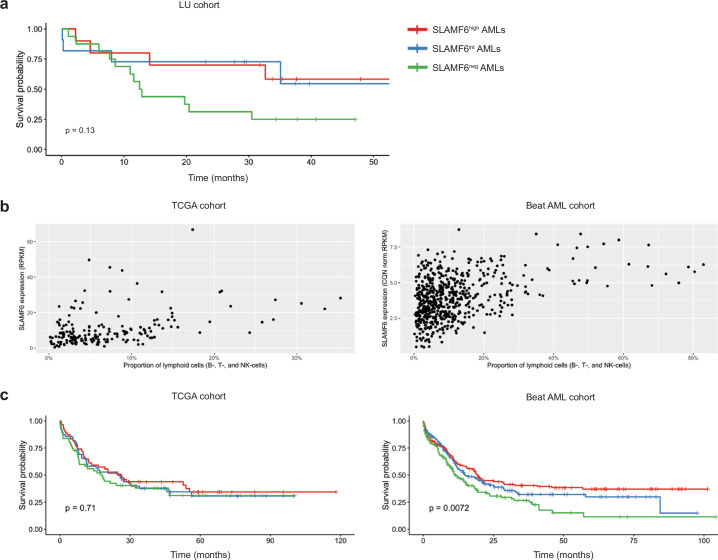
Correlation between SLAMF6 expression and outcome in AML. (**a**) Survival in the Lund cohort (n = 37 cases) based on SLAMF6 protein expression as determined by flow cytometry (log-rank test). (**b**) Lymphocyte content in the TCGA AML and Beat-AML gene expression datasets. (**c**) Survival in the TCGA AML (n = 164 cases) and Beat-AML (n = 455 cases) cohorts based on SLAMF6 gene expression after removal of cases with >15% lymphocyte content (log-rank test). Red: SLAMF6^High^ AML cases, blue: SLAMF6^Int^ AML cases, green: SLAMF6^Neg^ AML cases. Source Data

To characterize the expression of SLAMF6 in AML, we extended our analysis to include 50 participants with AML representing the major genetic subgroups (Supplementary Table 1). By flow cytometry, we found that 58% of AML samples were positive for SLAMF6, with high expression in 24% of cases and intermediate expression in 34% of cases (Fig. 2a). For the CD34^+^ AML cases, SLAMF6 expression was also assessed in the primitive CD34^+^CD38^low^ compartment, enriched for LSCs. Here, SLAMF6 was detected on 63% of the CD34^+^ AML samples, 37% with high and 25% with intermediate levels (Fig. 2b). Furthermore, SLAMF6 expression was not restricted to a specific genetic subtype but expressed in all major subtypes, including the poor prognostic groups with myelodysplasia-related mutations (AML-MR) and *TP53* mutations (Fig. 2c–f). SLAMF6 was also found to be expressed on 64% of cases with relapsed/refractory AML, where the prognosis is particularly dismal (Fig. 2g). Additionally, six of nine tested myeloid leukemia cell lines were found to be positive for SLAMF6 (Fig. 2h). There was no significant correlation between SLAMF6 protein expression and outcome in our cohort of 37 cases but a trend toward better survival for the cases expressing SLAMF6 (Extended Data Fig. 3a). SLAMF6 classification in large gene expression datasets is complicated by *SLAMF6* expression from infiltrating lymphocytes; however, after removal of 15 of 179 samples with high lymphocyte content by cell type deconvolution, we found no correlation between *SLAMF6* gene expression and outcome in The Cancer Genome Atlas (TCGA) cohort (Extended Data Fig. 3b, c). In the remaining 455 of 575 samples in the Beat-AML cohort, *SLAMF6* expression correlated with improved survival but all groups displayed 5-year survival rates of less than 50% (Extended Data Fig. 3b, c). Collectively, these analyses show no clear correlation between SLAMF6 status and survival, with relatively poor outcomes in both SLAMF6^+^ and SLAMF6^−^ AML. As SLAMF6 has been implicated in lymphocyte signaling in healthy tissues, we examined the co-expression pattern with other known immunomodulatory cell surface markers in our cohort. This analysis indicated a potential correlation with CD200, whereas no clear correlations were observed with either CD47, CD84, CD244 or PDL1 in this cohort (Fig. 2i–m). Taken together, these findings demonstrate that SLAMF6 is aberrantly expressed in a majority of AML cases across molecular and clinical classifications.

### SLAMF6 protects AML cells from T cell-mediated killing

**Fig. 3:**
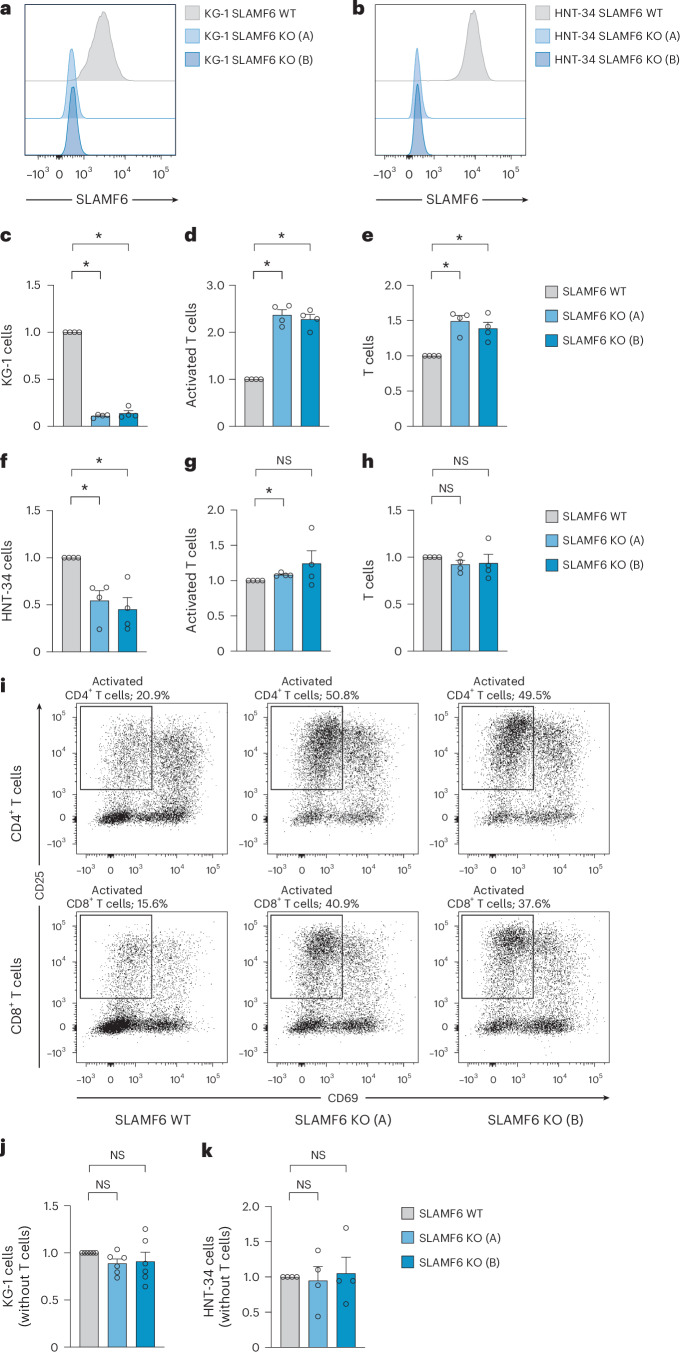
SLAMF6 inhibits T cell activation and killing. Expression of SLAMF6 by flow cytometry in wild-type and *SLAMF6*-knockout cells generated from the AML cell lines KG-1 (**a**) and HNT-34 (**b**) by CRISPR–Cas9. *SLAMF6* KO (A) and *SLAMF6* KO (B) denote *SLAMF6*-knockout cells generated by two different crRNAs. SLAMF6 WT denotes SLAMF6 wild-type cells mock-electroporated with a crRNA targeting the firefly luciferase gene. **c**–**e**, T cell-mediated killing of KG-1 cells (*P* = 0.029 for KO (A) and *P* = 0.029 for KO (B)) (**c**), frequency of activated T cells (*P* = 0.029 for KO (A) and *P* = 0.029 for KO (B)) (**d**) and total number of T cells (*P* = 0.029 for KO (A) and *P* = 0.029 for KO (B)) (**e**) in 72-h coculture assay with primary human T cells and wild-type and *SLAMF6*-knockout cells. Values were normalized to wild-type control. Data are shown as the mean ± s.e.m. (*n* = 4 T cell donors; two-sided Mann–Whitney *U*-test). **f**–**h**, T cell-mediated killing of HNT-34 cells (*P* = 0.029 for KO (A) and *P* = 0.029 for KO (B)) (**f**), frequency of activated T cells (*P* = 0.029 for KO (A) and *P* = 0.314 for KO (B)) (**g**) and total number of T cells (*P* = 0.314 for KO (A) and *P* = 0.029 for KO (B)) (**h**) in 72-h coculture assay with primary human T cells and wild-type and *SLAMF6*-knockout cells. Values were normalized to the wild-type control. Data are shown as the mean ± s.e.m. (*n* = 4 T cell donors; two-sided Mann–Whitney *U*-test). **i**, Representative T cell activation in coculture assay with KG-1, as determined by surface expression of CD25 and CD69 on CD4^+^ and CD8^+^ T cells by flow cytometry (one representative donor of four). **j**, Expansion of wild-type and *SLAMF6*-knockout KG-1 cells in the absence of T cells. Cell numbers after 72 h of culture were normalized to the wild-type control. Data are shown as the mean ± s.e.m. (*n* = 6 experiments; two-sided Mann–Whitney *U*-test, *P* = 0.054 for KO (A) and *P* = 0.372 for KO (B)). NS, not significant. **k**, Expansion of wild-type and *SLAMF6*-knockout HNT-34 cells in the absence of T cells. Cell numbers after 72 h of culture were normalized to the wild-type control. Data are shown as the mean ± s.e.m. (*n* = 6 experiments; two-sided Mann–Whitney *U*-test, *P* = 0.999 for KO (A) and *P* = 0.314 for KO (B)). Source Data

**Extended Data Fig. 4:**
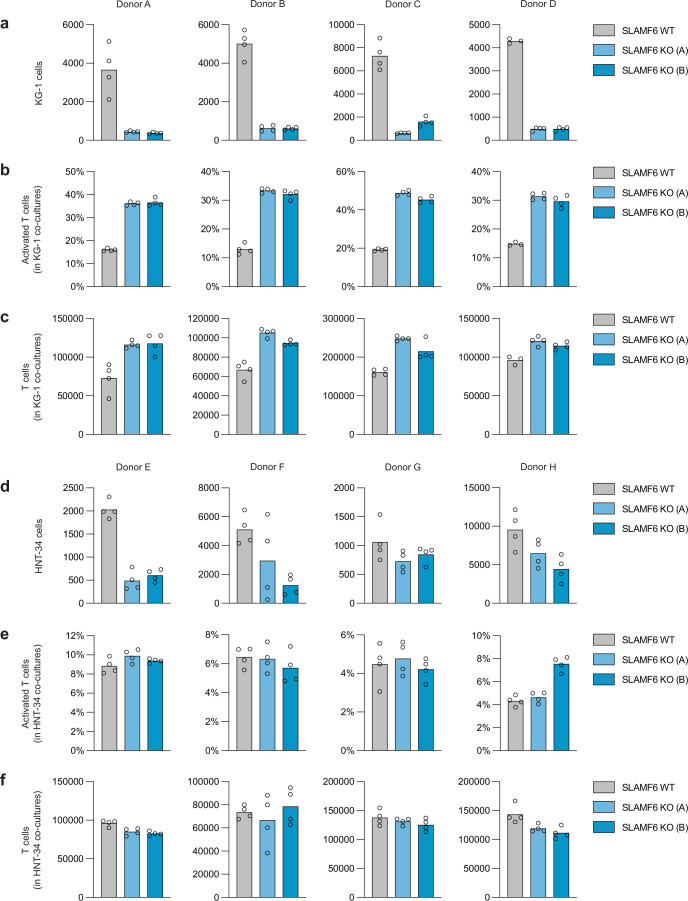
SLAMF6 inhibits T cell activation and killing of AML cells. (**a**) T cell-mediated killing of AML cells after 72 h of co-culture with primary human T cells and SLAMF6 wildtype and knockout KG-1 cells. (**b**) T cell activation in KG-1 co-cultures, as determined by surface expression of CD25 and CD69. (**c**) T cell numbers in KG-1 co-cultures. (**d**) T cell-mediated killing of AML cells after 72 h of co-culture with primary human T cells and SLAMF6 wildtype and knockout HNT-34 cells. (**e**) T cell activation in HNT-34 co-cultures, as determined by surface expression of CD25 and CD69. (**f**) T cell numbers in HNT-34 co-cultures. Data for individual donors for which the composite data is presented in Fig. 3c–h. Bars indicate mean values from four technical replicates for each donor and wildtype or knockout cell type. Source Data

**Extended Data Fig. 5:**
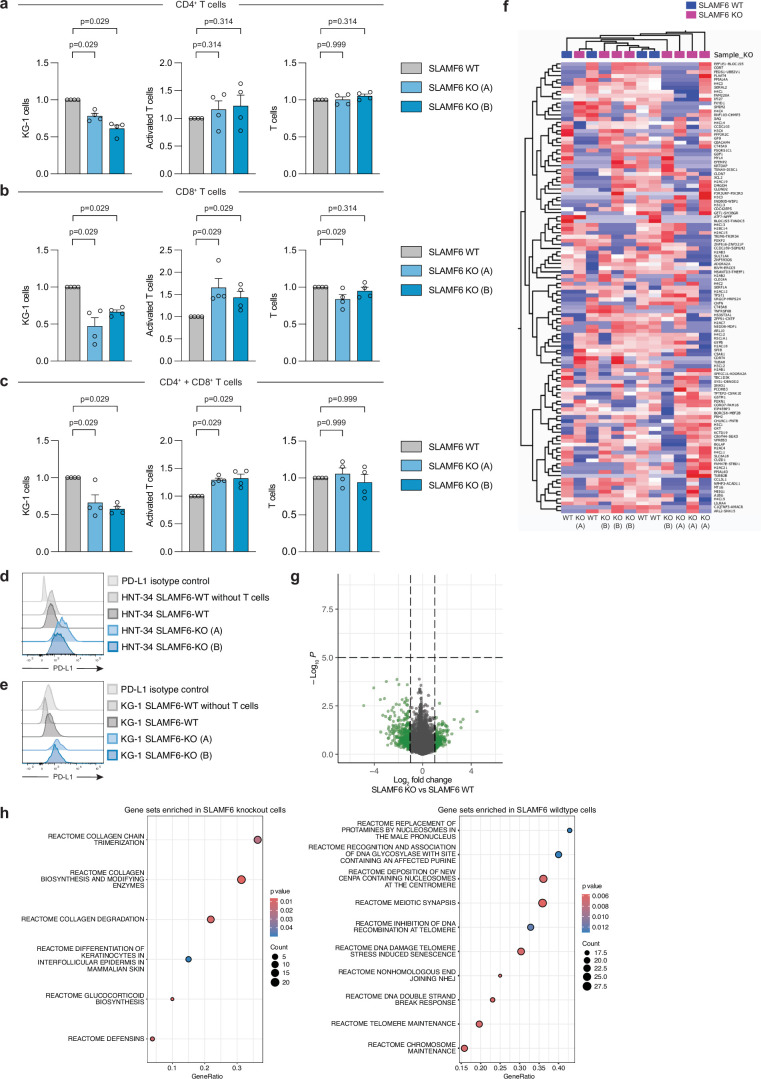
SLAMF6 inhibits killing by both CD4^+^ and CD8^+^ T cells but induces no major effects on gene expression in the absence of T cells. T cell activation and killing of SLAMF6 wildtype and knockout KG-1 AML cells in co-cultures with (**a**) CD4^+^ T cells, (**b**) CD8^+^ T cells and (**c**) both CD4^+^ and CD8^+^ T cells. Left: T cell-mediated killing of AML cells. Middle: Frequency of activated T cells based on expression of CD69 and CD25. Right: Total number of T cells. Values normalized to SLAMF6 wildtype control. Mean ± SEM of four T cell donors (two-sided Mann-Whitney U test). (**d**) PD-L1 expression after 72 h of co-culture with primary human T cells and SLAMF6 wildtype and knockout HNT-34 cells. One representative T cell donor out of the four in which co-culture with the T cells itself did not induce PD-L1. Six donors were tested in total. (**e**) PD-L1 expression after 72 h of co-culture with primary human T cells and SLAMF6 wildtype and knockout KG-1 cells. One representative T cell donor out of the two in which co-culture with the T cells itself did not induce PD-L1. Five donors were tested in total. (**f**) Heatmap with unbiased hierarchical clustering of both samples and genes. Samples include four replicates each of SLAMF6 wildtype HNT-34 cells and SLAMF6 knockout cells generated with the two guide RNA constructs ”A” and ”B”. Genes were filtered by variance >0.5, resulting in 115 differentially expressed genes. (**g**) Volcano plot of differentially expressed genes between the SLAMF6 wildtype and knockout cells (two-sided t-test, without multiple testing correction). Y axis represents significance denoted as the negative logarithm of the p value. None of the 19409 identified genes are significantly higher expressed in either group. Grey: Fold change between 0.5 and 2. Green: Fold change <0.5 or >2. (**h**) Gene sets enriched in SLAMF6 knockout (left) and wildtype (right) cells, based on gene set enrichment analysis (GSEA) using the Reactome database (fgsea test with multiple testing correction, threshold p < 0.05). Source Data

SLAMF6 signaling between neighboring T cells has been shown to dampen the T cell response^35–37^. Thus, we hypothesized that SLAMF6 expression on AML cells could mimic this mechanism and prevent T cell activation and killing. To test this, we knocked out the endogenous *SLAMF6* expression in KG-1 and HNT-34 AML cells by CRISPR–Cas9 using two different guide RNAs (Fig. 3a, b). We then assayed the killing of the wild-type and *SLAMF6*-knockout AML cells by primary T cells in an human leukocyte antigen (HLA) mismatch-driven coculture system. Knockout of *SLAMF6* in AML cells was found to render the cells highly susceptible to T cell-mediated killing, with an 88% decrease in the number of KG-1 cells after coculture (Fig. 3c and Extended Data Fig. 4a). The increase in T cell-mediated killing was accompanied by a substantial increase in the activation of both CD4^+^ and CD8^+^ T cells, as determined by surface expression of CD25 and CD69. On average, the proportion of activated T cells increased by 136% on the basis of two knockout constructs and four different T cell donors (Fig. 3d,i and Extended Data Fig. 4b). Consistent with this activation, the T cell population also expanded by 47% (Fig. 3e and Extended Data Fig. 4c). *SLAMF6* knockout in HNT-34 cells also generated a strong T cell response, which reduced the number of AML cells by 50% (Fig. 3f and Extended Data Fig. 4d). In the HNT-34 cocultures, T cell activation was only affected in certain donors, indicating that the effect on T cell activation is secondary to the effect on T cell-mediated killing (Fig. 3g,h and Extended Data Fig. 4e,f). T cell killing was also induced in cocultures with only CD4^+^ or CD8^+^ T cells, demonstrating that the effect is mediated by both cell types (Extended Data Fig. 5a–c). Furthermore, *SLAMF6* knockout increased PDL1 expression on the AML cells in cases where it was not already induced by the T cell coculture, suggesting potential interplay between the two pathways (Extended Data Fig. 5d,e). Lastly, we investigated whether the expression of *SLAMF6* has any direct effects on AML cells. RNA-seq of wild-type and *SLAMF6*-knockout HNT-34 AML cells revealed that the gene expression differences were too minute to separate wild-type and knockout cells by unsupervised hierarchical clustering (Extended Data Fig. 3f). Further analysis showed that no genes were significantly upregulated or downregulated by knockout of *SLAMF6* (Extended Data Fig. 3g) and gene set enrichment analysis (GSEA) did not identify any processes that could mediate the protective effect against T cell-mediated killing (Extended Data Fig. 3h). This absence of direct effects on the AML cells conforms with the established notion of SLAMF6 as a ligand whose predominant role is in regulating the activity of interacting lymphocytes. Additionally, knockout of *SLAMF6* did not affect AML cell survival or proliferation when cultured without T cells, confirming that SLAMF6 specifically protects AML cells from T cell elimination (Fig. 3j,k). In conclusion, these results demonstrate that SLAMF6 expression on AML cells mediates immune escape from T cell killing.

### SLAMF6^+^ AML cases display enrichment of naive T cells

**Fig. 4:**
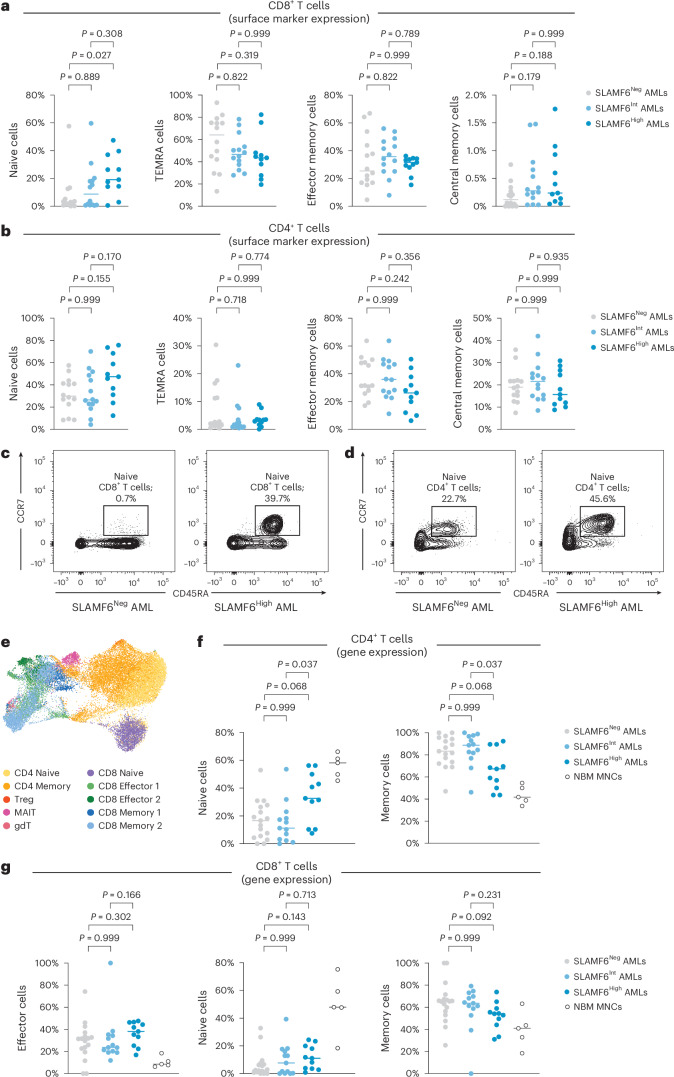
AML cases with SLAMF6 display altered T cell composition. **a**,**b**, Frequencies of naive, TEMRA, effector memory and central memory T cells for 39 participants with AML stratified by SLAMF6 expression, based on surface expression of CD45RA and CCR7 within the CD8^+^ T cell compartment (**a**) and the CD4^+^ T cell compartment (**b**) (Kruskal–Wallis test with Dunn’s post hoc test). Bars indicate median values. **c**,**d**, Frequencies of naive cells within the CD8^+^ (**c**) and CD4^+^ (**d**) T cell compartments for a participant with high SLAMF6 expression and a participant negative for SLAMF6, based on CD45RA and CCR7. **e**, UMAP and cell type classifications for the T cells from the same cohort of participants with AML based on gene expression profiling by scRNA-seq (28,932 cells from 41 cases). MAIT, mucosal-associated invariant T cell; gdT, gamma delta T cell. **f**,**g**, Frequencies of naive, effector and memory T cells within the CD4^+^ T cell compartment (**f**) and the CD8^+^ T cell compartment (**g**), based on single-cell gene expression signatures for each participant (*n* = 41 cases; Kruskal–Wallis test with Dunn’s post hoc test). Bars indicate median values. Source Data

**Extended Data Fig. 6:**
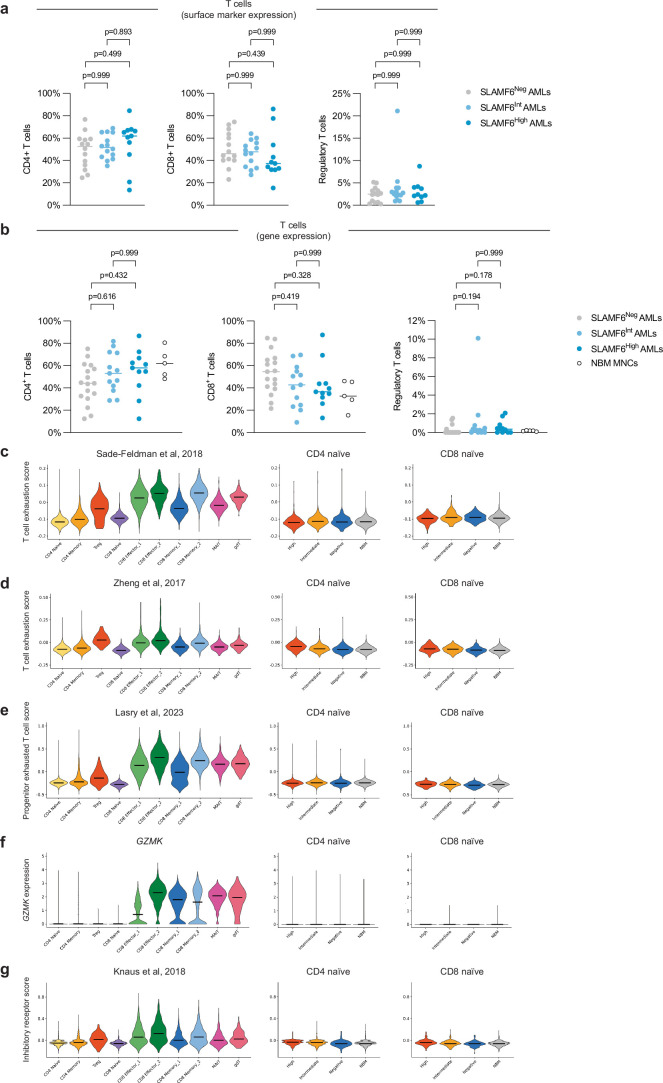
Naïve T cell populations enriched in SLAMF6^+^ AML do not display signs of T cell exhaustion. Frequencies of CD4^+^ T cells, CD8^+^ T cells and regulatory T cells within the total T cell population for each AML patient and NBM donor based on (**a**) surface protein expression by flow cytometry (n = 39 cases; Kruskal-Wallis test with Dunn’s post hoc test) and (**b**) global gene expression by single cell RNA sequencing (n = 41 cases; Kruskal-Wallis test with Dunn’s post hoc test). Similarity of T cell populations in primary AML samples to (**c**) exhausted T cells^46^, (**d**) exhausted T cells^47^ and (**e**) progenitor exhausted T cells^29^, based on global gene expression profiles generated by single cell RNA sequencing. (**f**) Expression of the progenitor exhausted T cell marker GZMK in T cell populations in primary AML samples. (**g**) Expression of inhibitory receptors upregulated in CD8 + T cells in AML patients^14^. Left: All T cell populations in all AML cases (n = 41 cases). Middle: The CD4^+^ naïve T cell population in AML cases classified as SLAMF6^High^ (red; n = 11 cases), SLAMF6^Int^ (yellow; n = 13 cases) and SLAMF6^Neg^ (blue; n = 17 cases) as well as CD4^+^ naïve T cells from normal bone marrow (grey). Right: The CD8^+^ naïve T cell population in AML cases and NBM. Violin plots with bars indicating median values. Y axis scales are consistent across left, middle and right panels. Source Data

To study whether SLAMF6 expression on AML cells affects T cell biology, we examined the T cell populations in 39 AML cases stratified by SLAMF6 expression. The proportions of CD4^+^, CD8^+^ and regulatory T cells were found to be unaffected by SLAMF6 expression on the leukemia cells (Extended Data Fig. 6a). However, AML cases with high SLAMF6 expression on the leukemia cells were found to have higher frequencies of CCR7^+^CD45RA^+^ T cells, an immunophenotype associated with naive cells. We observed a sevenfold difference in naive cell frequency in the CD8^+^ compartment and a trend toward higher frequency in the CD4^+^ compartment (Fig. 4a–d). To characterize these T cell populations in greater detail, we analyzed scRNA-seq data from the same AML cases and five NBM samples^45^ (Fig. 4e). Classification of individual T cells based on their gene expression profiles showed an enrichment of naive T cells in AML cases with high SLAMF6 expression and a corresponding decrease in memory T cells (Fig. 4f,g and Extended Data Fig. 6b). To further differentiate naive from exhausted T cells, we applied gene expression signatures of T cell exhaustion to our dataset and scored the cells for similarity to these profiles. In this analysis, the cells classified as naive exhibited the least similarity to both exhausted T cells^46,47^ (Extended Data Fig. 6c,d) and progenitor exhausted T cells (T_pex_)^29^ (Extended Data Fig. 6e). The cells were also found to be negative for the T_pex_ marker *GZMK* (Extended Data Fig. 6f). Importantly, there was no difference between the naive T cells from AML cases with and without SLAMF6 in any of these regards. Similarly, inhibitory receptors shown to be transcriptionally upregulated in CD8^+^ T cells from persons with AML^14^ were also lowly expressed in the naive T cell populations with no correlation with SLAMF6 expression (Extended Data Fig. 6g). These analyses indicate that the T cell landscape in SLAMF6^+^ AML is characterized by an enrichment of naive T cells and lower accumulation of memory T cells compared to other AML. This is consistent with a role of SLAMF6 in preventing T cell activation and inhibiting the antitumor T cell response.

### Antibody-mediated targeting of the SLAMF6 dimerization site inhibits the SLAMF6–SLAMF6 interaction

**Fig. 5:**
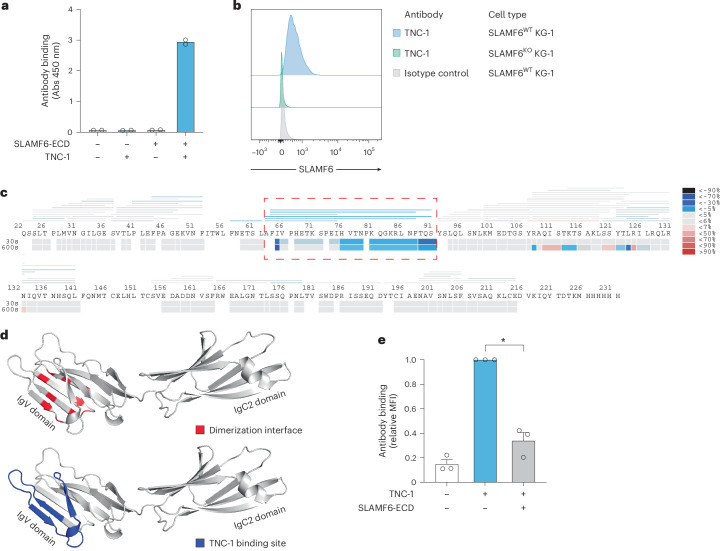
Antibody-mediated targeting of the SLAMF6 dimerization site inhibits the SLAMF6–SLAMF6 interaction. **a**, Binding of the SLAMF6 antibody TNC-1 to SLAMF6-ECD in ELISA. Bars indicate the mean values of two technical replicates. **b**, TNC-1 binding to wild-type and *SLAMF6*-knockout cells, generated by CRISPR–Cas9 in the AML cell line KG-1 with endogenous expression of SLAMF6. **c**, Mapping of the TNC-1 binding epitope in SLAMF6 by HDX-MS. The dashed red line denotes the determined epitope. The color scale denotes the change in epitope accessibility by incubation with antibody Fab fragment. **d**, Three-dimensional model of the SLAMF6 protein based on AlphaFold, with the SLAMF6–SLAMF6 dimerization interface marked in red and the TNC-1 binding site marked in blue. **e**, Binding of TNC-1 to SLAMF6 on primary T cells with and without preincubation of the cells with SLAMF6-ECD. Data are shown as the mean ± s.e.m. of three T cell donors, normalized to the MFI without preincubation for each donor (two-sided *t*-test with Welch’s correction, *P* = 0.010). Source Data

The strong immunosuppressive effect of the SLAMF6–SLAMF6 interaction between AML cells and T cells indicated that an antibody blocking this interaction could unleash a potent antitumor T cell response. Thus, we generated antibodies to SLAMF6 by phage-display technology. The candidate antibody, termed TNC-1, exhibited highly specific binding to SLAMF6, both in ELISA with the SLAMF6 extracellular domain (SLAMF6-ECD) (Fig. 5a) and in flow cytometry, where binding to endogenous SLAMF6 on the KG-1 cell line was completely ablated by *SLAMF6* knockout with CRISPR–Cas9 (Fig. 5b). The binding epitope in SLAMF6 was mapped by hydrogen–deuterium exchange (HDX)-MS to a part of the IgV domain that overlaps with the amino acid residues known to mediate the SLAMF6–SLAMF6 interaction (Fig. 5c, d and Supplementary Table 4)^48^. To functionally test whether the TNC-1 antibody could compete with SLAMF6 to block the interaction, binding of TNC-1 to primary T cells was assessed by flow cytometry with or without preincubation with soluble SLAMF6-ECD (Fig. 5e). The results showed that precoating the T cells with SLAMF6-ECD reduced TNC-1 binding, demonstrating competitive binding between TNC-1 and SLAMF6.

### SLAMF6 blockade induces T cell killing of AML cells in vitro

**Fig. 6:**
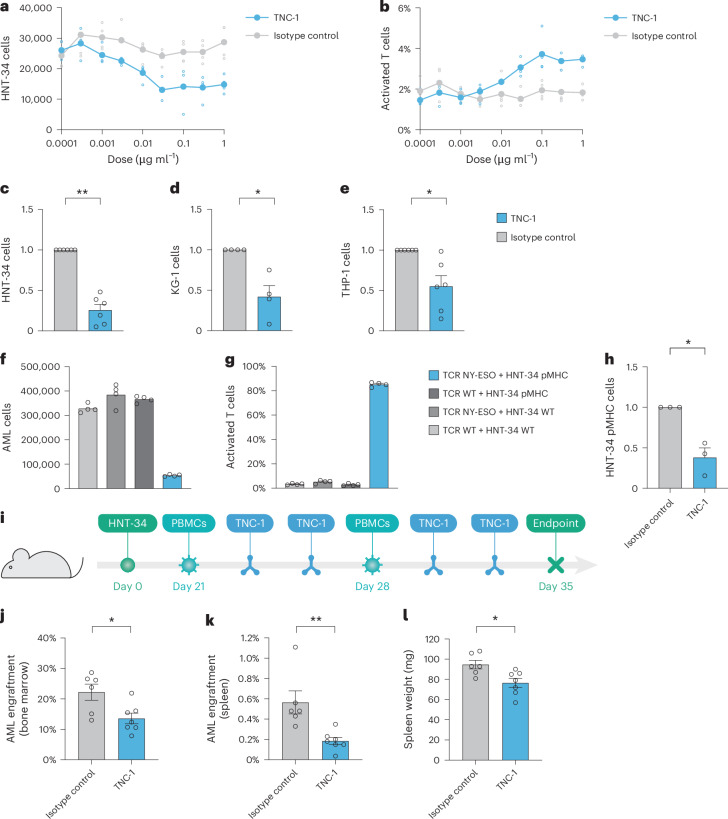
Antibody-mediated inhibition of SLAMF6 induces T cell activation and killing of AML cells. **a**, Dose-dependent killing of AML cells in a coculture assay with primary human T cells and HNT-34 AML cells, determined as the number of remaining AML cells after 72 h. Data are shown as the mean of four technical replicates per antibody and dose with one T cell donor. **b**, Dose-dependent T cell activation in the coculture assay, determined as the percentage of T cells positive for the activation markers CD25 and CD69 after 72 h. Data are shown as the mean of four technical replicates per antibody and dose with one T cell donor. **c**, T cell-mediated killing of HNT-34 cells in cocultures after treatment with 1 μg ml^−1^ antibody. Data are shown as the mean ± s.e.m. of six T cell donors, normalized to isotype control (two-sided Mann–Whitney *U*-test; *P* = 0.0022). **d**, T cell-mediated killing of KG-1 cells in cocultures after treatment with 1 μg ml^−1^ antibody. Data are shown as the mean ± s.e.m. of four T cell donors, normalized to isotype control (two-sided Mann–Whitney *U*-test; *P* = 0.029). **e**, T cell-mediated killing of THP-1 cells in cocultures after treatment with 1 μg ml^−1^ antibody. Data are shown as the mean ± s.e.m. of six T cell donors, normalized to the isotype control (two-sided Mann–Whitney *U*-test; *P* = 0.0022). **f**, T cell-mediated killing of AML cells by ectopic expression of the NY-ESO TCR and corresponding pMHC complex in 72 h cocultures with primary T cells and HNT-34 AML cells. Bars indicate the mean values of four technical replicates from one representative T cell donor of four. **g**, T cell activation induced by the matched expression of the NY-ESO TCR and pMHC complex in the coculture assay. Bars indicate the mean values of four technical replicates from one representative T cell donor of four. **h**, T cell-mediated killing of HNT-34 AML cells in cocultures with NY-ESO TCR and pMHC expression after treatment with 1 μg ml^−1^ antibody. Data are shown as the mean ± s.e.m. of three T cell donors, normalized to isotype control (*t*-test with Welch’s correction, *P* = 0.035). **i**, Schematic overview of the humanized mouse model for in vivo treatment with the SLAMF6 antibody TNC-1. Seven mice were treated with TNC-1 and six mice were treated with an isotype control antibody. **j**, AML engraftment in bone marrow after treatment, determined as the percentage of the total cell population constituted by human AML cells (two-sided Mann–Whitney *U*-test, *P* = 0.035). **k**, AML engraftment in spleen (two-sided Mann–Whitney *U*-test, *P* = 0.0023). **l**, Weights of isolated spleens (two-sided Mann–Whitney *U*-test, *P* = 0.013). Source Data

**Extended Data Fig. 7:**
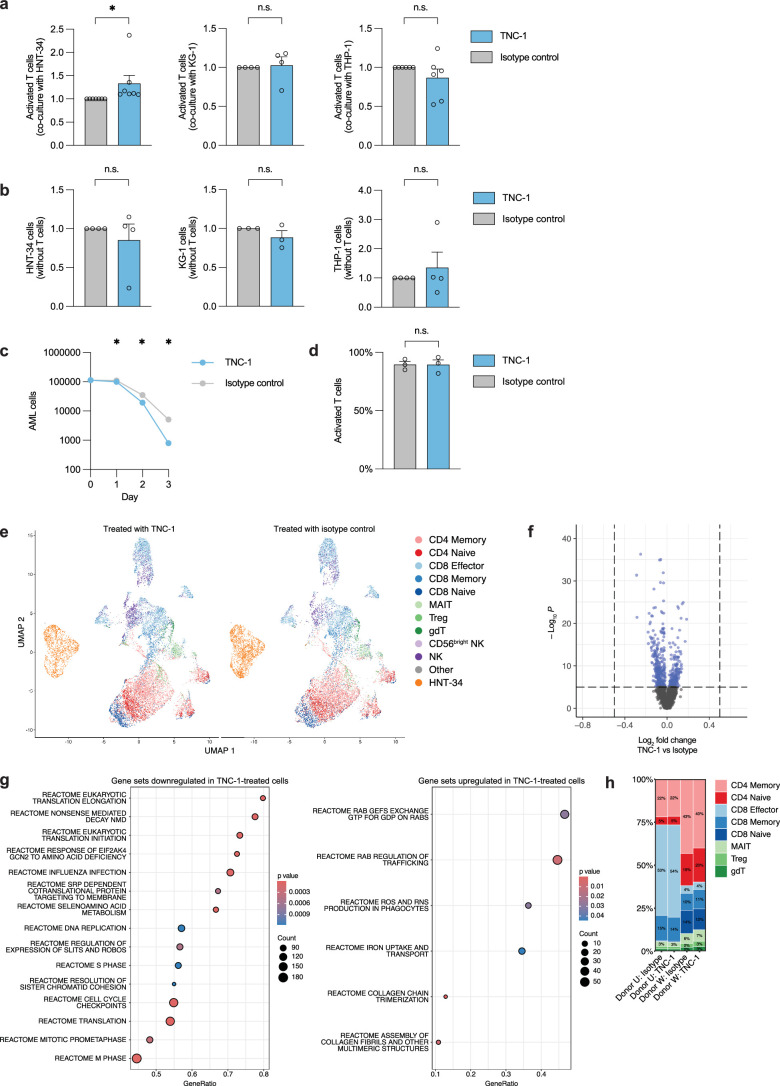
Treatment with TNC-1 induces T cell activation and gene expression changes associated with T cell-mediated killing. (**a**) T cell activation in response to in vitro treatment with TNC-1 in co-cultures with primary T cells and HNT-34 cells (left; p = 0.041), KG-1 cells (middle; p = 0.314) and THP-1 cells (right; p = 0.364), as determined by surface marker expression of CD25 and CD69. Mean ± SEM of four (KG-1) or six (HNT-34, THP-1) T cell donors, normalized to isotype control (two-sided Mann-Whitney U test). (**b**) AML cell killing in response to treatment with TNC-1 in suspension cultures without T cells, containing only HNT-34 cells (left; p = 0.999), KG-1 cells (middle; p = 0.700) and THP-1 cells (right; p = 0.999). Mean ± SEM of four experiments, normalized to isotype control (two-sided Mann-Whitney U test). (**c**) T cell-mediated killing of AML cells over time in co-cultures with T cells expressing the NY-ESO T cell receptor and HNT-34 AML cells expressing the corresponding peptide-MHC complex. Mean ± SEM of four technical replicates for each time point and treatment condition for one T cell donor (two-sided Mann-Whitney U test; p = 0.029 for all time points). (**d**) T cell activation after 72 h in the NY-ESO co-culture model, as determined by surface expression of CD25. Mean ± SEM of three T cell donors (two-sided Mann-Whitney U test; p = 0.999). (**e**) UMAP projection and cell type classifications for co-cultures with T cells and HNT-34 AML cells after treatment with TNC-1 (left; n = 16254 cells) or an isotype control antibody (right; n = 15723 cells), based on single-cell RNA sequencing. Joint projection for co-cultures with two independent T cell donors. MAIT: mucosal-associated invariant T cell, Treg: regulatory T cell, gdT: gamma delta T cell, NK: natural killer cell. (**f**) Volcano plot of differentially expressed genes between HNT-34 cells treated with TNC-1 or an isotype control antibody in T cell co-cultures (n = 23886 genes). Y axis represents significance denoted as the negative logarithm of the p value (two-sided t test, without multiple testing correction). Grey: P value > 10^−5^. Blue: P value < 10^−5^. (**g**) The 15 most significantly downregulated (left) and upregulated (right) gene sets in HNT-34 cells treated with TNC-1 compared to treatment with an isotype control antibody, based on gene set enrichment analysis (GSEA) using the Reactome database (fgsea test with multiple testing correction and threshold p < 0.05). (**h**) Distribution of T cell populations in two independent donors after treatment with TNC-1 or an isotype control antibody, based on single-cell RNA sequencing analysis. Source Data

**Extended Data Fig. 8:**
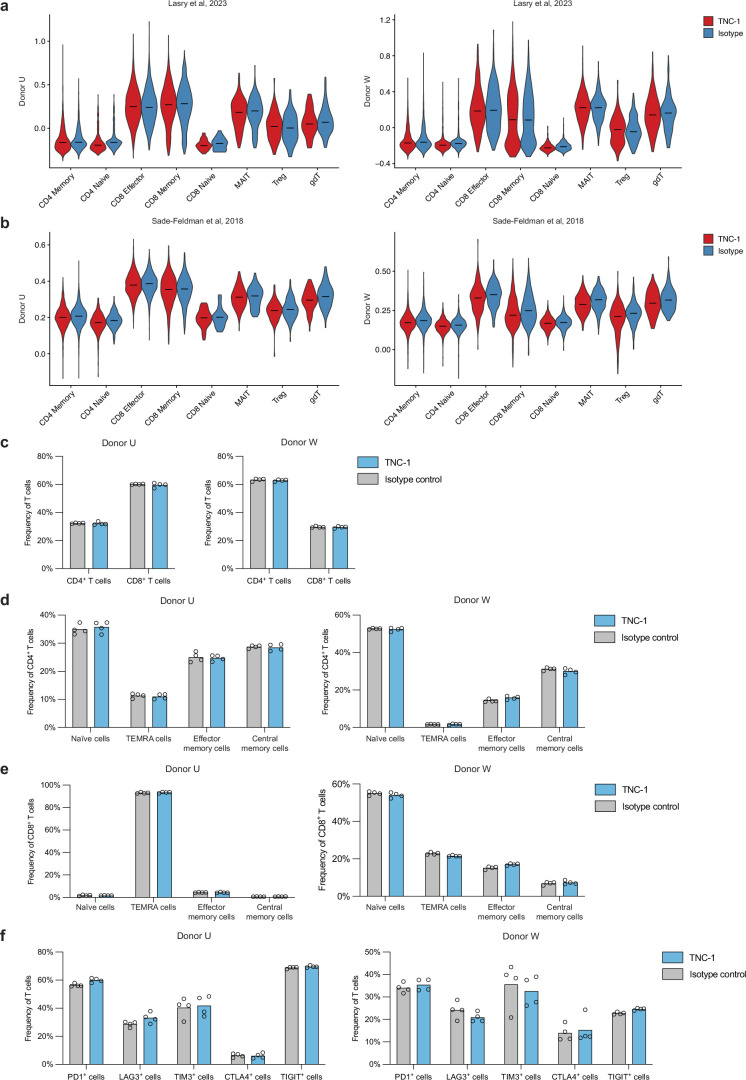
Treatment with TNC-1 does not induce major effects on T cell subset composition or exhaustion marker expression. Similarity of T cell populations to (**a**) progenitor exhausted T cells^29^ and (**b**) exhausted T cells^46^ after treatment of T cell and AML cell co-cultures with TNC-1 or an isotype control antibody for 72 h, based on gene expression profiles determined by single-cell RNA sequencing. Violin plots with bars indicating median values. Red: Distributions of T cells treated with TNC-1. Blue: Distributions of T cells treated with an isotype control antibody. (**c**) Frequencies of CD4^+^ and CD8^+^ T cells after treatment with TNC-1 in co-culture experiments with primary T cells and HNT-34 AML cells. (**d**) Composition of the CD4^+^ T cell population in co-cultures, based on surface expression of CD45RA and CCR7. (**e**) Composition of the CD8^+^ T cell population in co-cultures, based on surface expression of CD45RA and CCR7. (**f**) Expression of T cell inhibitory receptors and exhaustion markers after treatment with TNC-1. All data is based on surface marker profiling by flow cytometry. Mean of four technical replicates for each donor and treatment condition. Source Data

**Extended Data Fig. 9:**
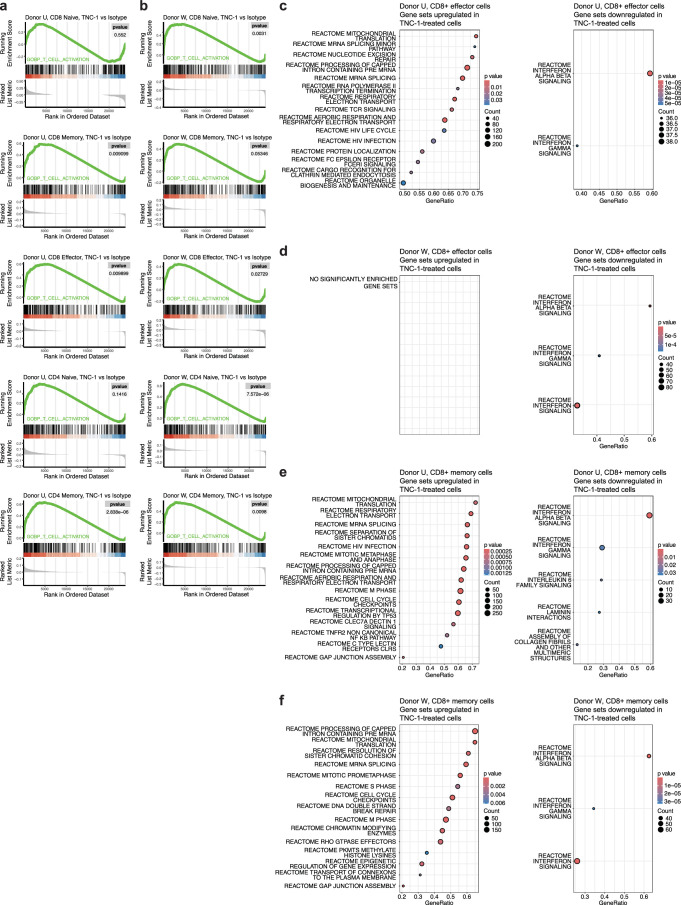
Treatment with TNC-1 induces gene expression changes in CD8^+^ effector and memory T cells associated with an enhanced T cell response. Increased expression of genes associated with T cell activation in T cell populations from (**a**) Donor U and (**b**) Donor W after treatment with TNC-1 compared to an isotype control antibody, based on gene set enrichment analysis of single-cell RNA sequencing data. (**c**) The 15 most significantly upregulated (left) and downregulated (right) gene sets in cells classified as CD8^+^ effector T cells after treatment of co-cultures containing primary T cells from Donor U and HNT-34 cells with TNC-1 compared to an isotype control antibody, based on gene set enrichment analysis (GSEA) of single-cell RNA sequencing data using the Reactome database. (**d**) Gene sets upregulated (left) and downregulated (right) in CD8^+^ effector T cells from Donor W after treatment of co-cultures with TNC-1. (**e**) Gene sets upregulated (left) and downregulated (right) in CD8^+^ memory T cells from Donor U after treatment of co-cultures with TNC-1. (**f**) Gene sets upregulated (left) and downregulated (right) in CD8^+^ memory T cells from Donor W after treatment of co-cultures with TNC-1. All p values are based on the fgsea test with correction for multiple testing. Gene sets are included with a significance threshold of p < 0.05. Source Data

To determine the functional effects of the SLAMF6 antibody, we performed treatment experiments in cocultures with AML cells and primary T cells. To ensure that the effects of the antibody would be limited to disruption of the SLAMF6–SLAMF6 interaction, the antibody was constructed as LALA-mutant hIgG1, thus preventing Fc receptor binding and induction of antibody-dependent cellular cytotoxicity (ADCC), antibody-dependent cellular phagocytosis (ADCP) and complement-dependent cytotoxicity (CDC). TNC-1 was found to induce T cell-mediated killing of HNT-34 AML cells in a dose-dependent manner with a corresponding increase in T cell activation (Fig. 6a,b). Treatment of additional SLAMF6^+^ AML cell lines with the antibody generated consistent induction of T cell-mediated killing and reduced the number of HNT-34 cells by 75%, KG-1 cells by 58% and THP-1 cells by 45% (Fig. 6c–e), with different degrees of T cell activation (Extended Data Fig. 7a). No reduction in cell number was observed in the absence of T cells, confirming that the cell killing is mediated by effector cells (Extended Data Fig. 7b). To assess the effect of the TNC-1 antibody in an HLA-matched model, we established a coculture system with HNT-34 AML cells transduced with a peptide–major histocompatibility complex (pMHC) complex and primary T cells transduced with the NY-ESO T cell receptor (TCR) that is reactive against the pMHC complex. The TCR–pMHC combination produced very strong effects on T cell activation and killing (Fig. 6f,g). Nevertheless, treatment with the TNC-1 antibody further increased T cell-mediated killing and reduced the number of AML cells by 62% (Fig. 6h and Extended Data Fig. 7c). As the TCR–pMHC combination itself induced activation of 90% of T cells, further stimulation by the antibody could not be observed (Extended Data Fig. 7d). To characterize the effect of the antibody in greater detail, we performed scRNA-seq on T cell and HNT-34 AML cell cocultures after treatment with TNC-1 (Extended Data Fig. 7e). The impact on the AML cells was consistent with the increase in T cell-mediated killing. The strongest effect was the downregulation of genes typically expressed in functional proliferative cells such as those involved in cell division, protein translation and macromolecule biosynthesis. Upregulated gene sets included production of reactive oxygen species, which is increased during T cell-induced apoptosis (Extended Data Fig. 7f,g). The T cells were classified on the basis of established gene expression signatures of major subpopulations^49^, which showed the distribution of naive, effector and memory T cells to be unaffected by TNC-1 treatment (Extended Data Fig. 7h). There were also no considerable changes in T cell exhaustion (Extended Data Fig. 8a,b). This was confirmed by analysis of surface marker expression (Extended Data Fig. 8c–f). However, both CD8^+^ effector cells and almost all other T cell populations were found to have gene expression signatures indicating increased activation (Extended Data Fig. 9a,b). This was supported by global GSEA, which identified TCR signaling and gene signatures associated with T cell expansion as the most significantly upregulated in CD8^+^ effector T cells from donor U upon TNC-1 treatment. Downregulated gene sets contained interferon-inducible genes, likely indicating that the early interferon response had subsided as the T cell response progressed (Extended Data Fig. 9c). For donor W, no gene sets were significantly upregulated in CD8^+^ effector T cells, likely because of the T cell response already receding after near elimination of the AML cells (Extended Data Fig. 9d). Similarly to effector cells, CD8^+^ memory T cells responded to TNC-1 treatment with increased expression of genes associated with T cell expansion and nuclear factor κB signaling, indicative of an enhanced memory T cell response (Extended Data Fig. 9e,f). In summary, these results demonstrate that treatment with an SLAMF6-blocking antibody can induce T cell-mediated killing of AML cells.

### The SLAMF6-blocking antibody TNC-1 induces T cell killing in vivo

**Fig. 7:**
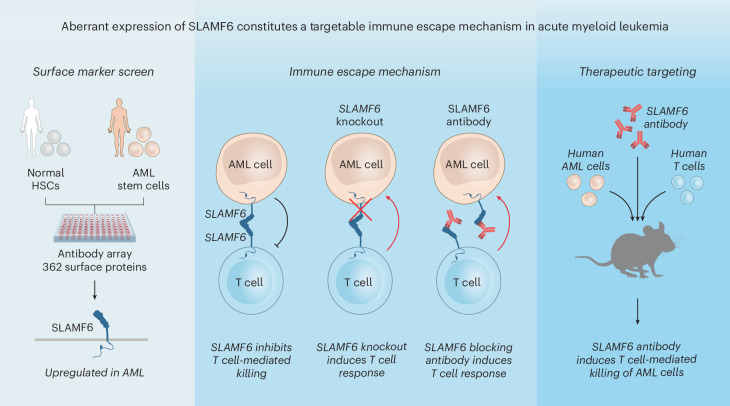
Graphical abstract. Left, SLAMF6 is upregulated in 60% of AML cases but not expressed on healthy HSPCs. Middle, SLAMF6 mediates protection against T cell-mediated killing. Genetic knockout or blocking antibodies reverse this effect and unleash a potent antileukemia T cell response. Right, the SLAMF6-blocking antibody TNC-1 induces a strong and specific immune response that reduces leukemia burden in humanized mice while sparing healthy stem and progenitor cells.

**Extended Data Fig. 10:**
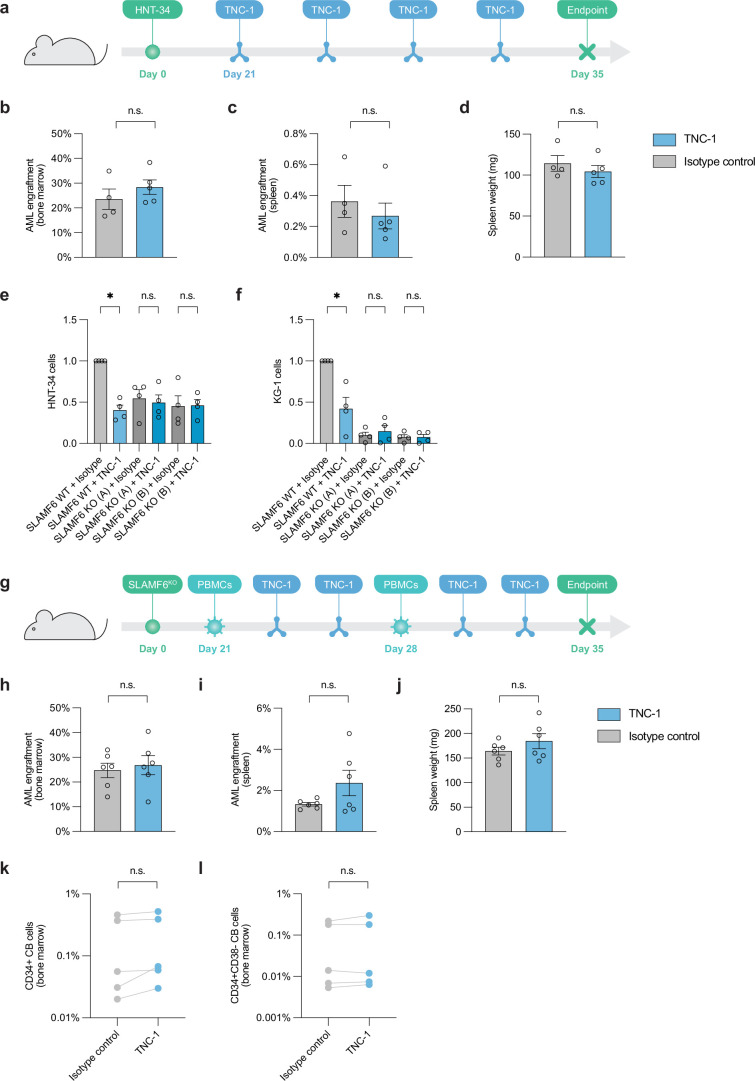
Treatment with TNC-1 does not induce T cell-mediated killing of SLAMF6 knockout AML cells or normal hematopoietic stem and progenitor cells. (**a**) Schematic overview of the humanized mouse model without human PBMCs. Five mice were treated with TNC-1 and four mice were treated with an isotype control antibody (all statistical testing performed with two-sided Mann-Whitney U test). (**b**) AML engraftment in bone marrow after treatment with TNC-1 following transplantation of HNT-34 AML cells without co-transplantation of human PBMCs, determined as the percentage of the total cell population constituted by human AML cells (p = 0.413). (**c**) AML engraftment in spleen (p = 0.413). (**d**) Weights of isolated spleens (p = 0.413). (**e**) T cell-mediated killing of HNT-34 cells in reponse to treatment with TNC-1 in 72 h co-culture experiments with primary T cells and SLAMF6 wildtype and knockout AML cells. Mean ± SEM of four T cell donors, normalized to SLAMF6 wildtype and isotype control (two-sided Mann-Whitney U test; p = 0.029 for WT, p = 0.886 for KO (A), p = 0.886 for KO (B)). (**f**) T cell-mediated killing of KG-1 cells in T cell co-culture assay. Mean ± SEM of four T cell donors, normalized to SLAMF6 wildtype and isotype control (two-sided Mann-Whitney U test; p = 0.029 for WT, p = 0.886 for KO (A), p = 0.886 for KO (B)). (**g**) Schematic overview of the mouse model with SLAMF6 knockout HNT-34 cells. Six mice were treated with TNC-1 and six mice were treated with an isotype control antibody (all statistical testing performed with two-sided Mann-Whitney U test). (**h**) AML engraftment in bone marrow after treatment with TNC-1 following transplantation of primary human T cells and SLAMF6 knockout HNT-34 cells, determined as the percentage of the total cell population constituted by human AML cells (p = 0.818). (**i**) AML engraftment in spleen (p = 0.589). (**j**) Weights of isolated spleens (p = 0.485). Bone marrow engraftment of (**k**) CD34^+^ cells (Wilcoxon signed-rank test; p = 0.063) and (**l**) CD34^+^CD38^low^ cells (Wilcoxon signed-rank test; p = 0.625) in response to treatment with TNC-1 after transplantation of CD34^+^ cells from human umbilical cord blood. For each of five different cord blood donors, one mouse was treated with TNC-1 and one with an isotype control antibody. Connecting lines indicate mice engrafted with cells from the same donor. Source Data

To evaluate the effect of the TNC-1 antibody in vivo, we established a humanized mouse model wherein human HNT-34 AML cells and human peripheral blood mononuclear cells (PBMCs) were sequentially cotransplanted into irradiated immunodeficient NSG-S mice. Mice were subsequently treated with either TNC-1 or an isotype-matched control antibody twice weekly starting immediately after each PBMC transplantation, for a total of four doses over 2 weeks (Fig. 6i). The antibody treatment resulted in a reduction in the leukemia burden in the bone marrow by 50% (Fig. 6j). TNC-1 also induced a marked antileukemic effect in the spleen with a reduction in leukemic engraftment by 63% (Fig. 6k) and a decrease in overall spleen weight (Fig. 6l). To confirm that the antibody effect is dependent on human effector cells, we also performed an identical treatment experiment without cotransplantation of human PBMCs. In this setting, there was no effect on tumor burden, demonstrating that the antibody acts through the antitumor immune response (Extended Data Fig. 10a–d). Furthermore, the lack of cross-reactivity to murine SLAMF6 ensured that the effect was mediated by human effector cells. Next, we performed antibody treatment experiments with *SLAMF6*-knockout cells to assess whether the antibody effect requires SLAMF6 expression on the AML cells. In the in vitro coculture system, the TNC-1 antibody was found to induce killing of SLAMF6^+^ but not SLAMF6^−^ cells in both HNT-34 and KG-1 models (Extended Data Fig. 10e,f). Accordingly, in vivo treatment of *SLAMF6*-knockout cells with TNC-1 did not induce T cell-mediated killing of HNT-34 AML cells (Extended Data Fig. 10g–j). This suggests that AML cells expressing SLAMF6 are particularly sensitive to therapeutic targeting of the SLAMF6 immune checkpoint. Consistent with these results, TNC-1 did not affect the levels of healthy HSPCs in vivo (Extended Data Fig. 10k,l), suggesting that SLAMF6 blockade could be a specific and well-tolerated treatment strategy. In conclusion, these results demonstrate that an antibody blocking the SLAMF6–SLAMF6 interaction can counteract SLAMF6 upregulation and elicit a strong antitumor T cell response both in vitro and in vivo (Fig. 7).

## Discussion

In this study, we describe a previously unknown immune escape mechanism in cancer and show that it can be targeted with therapeutic antibodies. We show that the lymphocyte marker SLAMF6 is unexpectedly aberrantly expressed on primitive AML cells and demonstrate that the SLAMF6 upregulation protects AML cells from T cell-mediated killing. We also show that a new antibody targeting the dimerization interface on SLAMF6 can break the immunosuppressive SLAMF6–SLAMF6 interaction between AML cells and T cells and unleash a T cell response that eliminates AML cells both in vitro and in vivo.

Despite advances in chemotherapy and the emergence of several targeted therapies, including specific inhibitors of FLT3, IDH1/2 and BCL-2, AML remains a highly lethal disorder and new treatments are greatly needed^50^. To identify novel drug targets in AML, we performed an arrayed antibody screen and found SLAMF6 to be aberrantly expressed in the majority of AML cases (25/42, 60%). This is the first study to demonstrate aberrant expression of SLAMF6 in any type of cancer. The cases include the AML-MR subtype and mutations in *TP53*, which have the most dismal prognoses and respond poorly to current treatments. We also characterized the expression of SLAMF6 on healthy cells, which was found to be highly restricted. SLAMF6 is known to only be expressed in the hematopoietic system^51^ and we here show that the expression is limited to T cells, B cells, NK cells and eosinophils. We also confirm the established notion that SLAMF6 is not expressed on healthy monocytes, despite a recent suggestion to the contrary^52^. Notably, SLAMF6 is not expressed on healthy HSPCs. The on-target off-tumor activity of an antibody targeting SLAMF6 is, therefore, likely to be limited and clinically manageable. This distinguishes SLAMF6 from many existing AML targets such as CD33, CD47, CD96, CD123, C-type lectin-like molecule 1 and TIM3, which are all expressed to various degrees on HSPCs^2^. The selective expression also makes SLAMF6 an attractive marker for separating LSCs from hematopoietic stem cells and could potentially be used to track the leukemia cell population over time.

As HSPCs do not express SLAMF6, it is not present on the cell of origin in AML but must instead be upregulated during leukemogenesis. However, this is unlikely to be a direct effect of a particular driver mutation as we show that SLAMF6 is expressed in all major subtypes and not correlated with any specific genetic aberrations. Thus, upregulation of SLAMF6 more likely derives from selective pressure to avoid elimination by antitumor T cells. The fact that a majority of AML cases upregulate this previously silenced gene indicates that there is strong evolutionary pressure to escape T cell recognition and expression of SLAMF6 confers a substantial selective advantage in this environment.

Furthermore, we show that knocking out endogenous *SLAMF6* in AML cells by CRISPR–Cas9 induces T cell activation and killing, demonstrating that *SLAMF6* upregulation is an immune evasion mechanism that protects AML cells from T cell-mediated killing. Although SLAMF6 was initially proposed as a costimulatory receptor for the TCR, recent work suggests that the role might be inhibitory rather than stimulatory^34–37,53–60^. Ectopic overexpression of *SLAMF6* in melanoma cells has been shown to reduce the activation of interacting T cells in vitro and to promote the expansion of melanoma cells in the presence of adoptively transferred T cells in vivo^35,37^. Our study is the first to examine the role of endogenous human SLAMF6 on tumor cells. We show not only that SLAMF6 inhibits T cell activation but also that AML cells depend on SLAMF6 for avoiding elimination by T cells. This conforms with work in the murine system, where knockout of *SLAMF6* (Ly108) on both T cells and antigen-presenting cells leads to increased T cell activation^37^. However, the work in *SLAMF6*-knockout mice has produced conflicting results in terms of both frequency and effector functions of CD8^+^ T cells^36,37^. Translation from mouse to human is further complicated by the considerably different expression patterns of *SLAMF6*. Whereas the expression in humans is limited to T cells, B cells, NK cells and eosinophils, murine *SLAMF6* is also expressed on neutrophils and all HSPC populations^61^. *SLAMF6* knockout would, thus, be expected to induce distinct phenotypes in humans and in mice, emphasizing the importance of further studies in human and humanized models.

The identification of aberrant *SLAMF6* expression as an immune escape mechanism in 60% of AML cases supports the notion that AML requires extensive immune evasion to avoid the immune surveillance in the bone marrow and that upregulation of *SLAMF6* is one of the most common mechanisms whereby this is achieved. In addition to SLAMF6, multiple cell surface proteins normally expressed on T cells have been shown to be aberrantly expressed in AML. For example, CD2, CD5, CD7, CD25, CD200 and TIM3 are all present on different subsets of persons with AML^62^. Although these proteins perform distinct functions, their upregulation suggests that AML cells can adopt T cell transcriptional programs and that T cell mimicry may contribute to leukemogenesis through different mechanisms.

In this study, we did not find a clear correlation between SLAMF6 expression and treatment response across our cohort and the larger TCGA and Beat-AML datasets. This is probably because of the fact that these persons have been treated with therapies that act independently of the anticancer immune response. However, SLAMF6 expression would likely dictate the response to treatments targeting the SLAMF6 checkpoint and potentially also to other immunotherapies that depend on T cell activity.

Consistent with its immunosuppressive role, we found that SLAMF6 expression on AML cells largely predicts T cell composition. Persons with AML generally have elevated levels of effector and memory T cells in the bone marrow as the result of an inflammatory antileukemia response^63^. However, cases with high SLAMF6 expression were found to have significantly higher proportions of naive T cells in both the CD4^+^ and CD8^+^ compartments compared to cases without SLAMF6. These findings suggest that SLAMF6 prevents T cell activation in these cases and that inhibition of the SLAMF6–SLAMF6 interaction could unleash a potent antitumor immune response.

To block the immunosuppressive SLAMF6–SLAMF6 interaction and restore antitumor T cell activity, we generated SLAMF6 antibodies by phage-display technology and identified an antibody targeting the dimerization interface. We demonstrate that this antibody breaks the interaction and enables T cell activation and killing of AML cells both in vitro and in humanized in vivo models. SLAMF6 antibodies have previously been suggested as potential cancer therapeutics but through different mechanisms. The inherent expression of SLAMF6 on B cells has generated interest in SLAMF6 as a tumor cell marker on malignancies of the B cell lineage, in a mode similar to CD19 and CD20. Accordingly, antibodies to SLAMF6 have been shown to induce killing of human B cell leukemia cells. However, these antibodies are capable of binding to Fc receptors and mediate killing through ADCC, ADCP and CDC^36,60,64^. In the present study, we instead used a LALA-mutant hIgG1 antibody, which does not bind to Fc receptors on effector cells but mediates its effect exclusively through binding to the SLAMF6 dimerization site. Thus, this is the first study to demonstrate that breaking the SLAMF6 interaction is sufficient to induce T cell activation and killing of tumor cells. In addition to antibodies, treatment with the soluble ectodomain of SLAMF6 has been shown to induce activation and expansion of antimelanoma T cells but the overlap between the two mechanisms remains to be determined^35^.

In conclusion, we describe a completely novel immune escape mechanism in cancer, in which AML cells induce aberrant expression of the lymphocyte marker SLAMF6 and thereby inhibit T cell-mediated killing, in a process analogous to the PDL1–PD1 interaction. Furthermore, we show that a novel fully human antibody targeting the SLAMF6 dimerization interface can block the immunosuppressive effect and unleash a potent antitumor T cell response both in vitro and in humanized in vivo models. Altogether, these findings present a new strategy for cancer immunotherapy in AML, where novel treatments are highly needed.

## Methods

### Ethics

The research complied with all relevant ethical regulations. Primary samples were collected at Skåne University Hospital after written informed consent from participants and in accordance with the Declaration of Helsinki. Experiments with primary leukemia samples were approved by the Swedish Ethical Review Authority (DNR 2023-01550). Aspects of the study involving research animals were conducted in accordance with local ethical regulations and approved by the regional Animal Ethics Committee of Malmö/Lund (7071/2020).

### Primary cells and cell lines

Bone marrow aspirates and peripheral blood samples were collected from healthy controls and participants with AML or myelodysplastic syndrome. Sample sizes were not predetermined by statistical methods but chosen on the basis of sample availability. Healthy bone marrow donors received modest financial compensation. No other compensation was awarded for study participation. Mononuclear cells were isolated by gradient separation using Lymphoprep (GE Healthcare) and viably frozen. Genetic profiling was performed by whole-exome sequencing and RNA-seq as previously described^45^. Cases were genetically classified according to the European LeukemiaNet (ELN) 2022 (ref. ^65^), International Consensus Classification (ICC) 2022 (ref. ^66^) and World Health Organization 2022 (ref. ^67^) guidelines as part of standard clinical practice, based on targeted sequencing. Participants included in the study and their clinical characteristics are shown in Supplementary Table 1. Primary leukemia samples were collected from both male and female participants to allow for equitable implementation of clinically relevant findings. The CMK, KG-1, HNT-34, K562, MonoMac6, NB4, OCI-AML3, TF-1 and THP-1 cell lines (Leibniz Institute German Collection of Microorganisms and Cell Cultures (DSMZ)) were cultured in RPMI medium (Thermo Fisher) with penicillin–streptomycin (Cytiva) and varying concentrations of FBS (Cytiva) and granulocyte-macrophage colony-stimulating factor (GM-CSF; Peprotech) according to the provider’s recommendations. T cells transduced with NY-ESO were cultured in AIM V medium (Thermo Fisher) with 10% FBS (Cytiva), 10 mM HEPES (Thermo Fisher), 1 mM sodium pyruvate (Thermo Fisher), penicillin–streptomycin (Cytiva) and 30 U per ml recombinant human interleukin 2 (hIL-2; R&D Systems). All cell lines were authenticated by genotyping, last performed in July 2024 (Eurofins Scientific).

### Cell surface marker screen

An arrayed antibody screen with 362 PE-conjugated antibodies (Supplementary Table 2) was performed using the LEGENDScreen platform (BioLegend, 700011). The screen was supplemented by antibodies to CD3, CD19, CD34 and CD38, as well as the viability marker 7-AAD (BD Biosciences). Antibodies and reagents are listed in Supplementary Table 3. Bone marrow mononuclear cells from participants with AML with *TP53* mutations (*n* = 3) and healthy controls (*n* = 3) were stained at 4 °C for 20 min, followed by washing and resuspension for flow cytometry analysis on an LSR Fortessa II (BD Biosciences). The gating strategy for SLAMF6 detection is described in Supplementary Fig. 1. The MFI and percentage of positive cells based on isotype controls for each marker within the 7-AAD^−^CD3^−^CD19^−^CD34^+^CD38^low^ fractions were used to compare the expression in AML and NBM. Highly expressed markers in the 7-AAD^−^CD3^−^CD19^−^CD34^+^CD38^low^ NBM population (MFI > 10,000 or >10% positive cells) were excluded. Analyzed cell surface markers were scored according to the differential expression in AML and NBM based on fold change (AML^MFI^/NBM^MFI^) and absolute shift (AML^MFI^ − NBM^MFI^). Candidates were then ranked on the basis of the average of their ranks in the two analyses and presented with the difference in MFI between AML and NBM (AML^MFI^ − NBM^MFI^).

### SLAMF6 expression on AML samples and healthy cells

Leukocyte concentrates from peripheral blood from healthy donors were lysed with ammonium chloride (StemCell Technologies), viably frozen and thawed before staining with 7-AAD and combinations of the following antibodies: CD3–PE/Cy7 (BioLegend, 344816), CD7–BV711 (BioLegend, 564018), CD11c–BV711 (BioLegend, 301630), CD14–BV421 (BioLegend, 301830), CD16–APC (BioLegend, 302012), CD19–APC/Cy7 (BioLegend, 302218), CD56–AF488 (BioLegend, 318312), CD123–AF488 (BioLegend, 306036), HLA-DR–APC (BioLegend, 307610) and either SLAMF6–PE (BioLegend, 317208) or PE-conjugated mIgG1 isotype control (BioLegend, 400114). Monocytes were defined as FSC-A^high^SSC-A^int^CD3^−^CD19^−^CD7^−^CD14^+^. Granulocytes were defined as FSC-A^high^SSC-A^high^SSC-H^int^CD16^high^. Eosinophils were defined as FSC-A^high^SSC-A^high^SSC-H^high^CD16^low^. Dendritic cells were defined as SSC-A^low/int^CD3^−^CD19^−^CD14^−^HLA-DR^+^. B cells were defined as FSC-A^low^SSC-A^low^CD3^−^CD19^+^. T cells were defined as FSC-A^low^SSC-A^low^CD19^−^CD3^+^. NK cells were defined as FSC-A^low^SSC-A^low^CD19^−^CD3^−^CD14^−^CD7^+^. Mononuclear cells from AML samples were thawed and stained with 7-AAD (BD Biosciences) and the following antibodies: CD3–PE/Cy7 (BioLegend, 344816), CD19–APC/Cy7 (BioLegend, 302218), CD34–AF488 (BioLegend, 343518), CD38–BV711 (BioLegend, 303528) and either SLAMF6–PE (BioLegend, 317208) or PE-conjugated mIgG1 isotype control (BioLegend, 400114). Flow cytometry was performed using an LSR Fortessa II (BD Biosciences). SLAMF6 expression was defined on the basis of the extent of the flow cytometric shift between signals with the SLAMF6 antibody and the isotype control antibody. Shifts with >50% of the cells outside the isotype peak were defined as ‘high’, 10–50% was defined as ‘intermediate’ and <10% was defined as ‘negative’.

### T cells and immunomodulatory markers in AML samples

Mononuclear cells from AML samples were thawed and stained with 7-AAD (BD Biosciences) and combinations of the following antibodies: CD96–BV421 (BioLegend, 338418), IL1RAP–BV421 (BD, 748107), CD38–BV711 (BioLegend, 303528), CD34–AF488 (BioLegend, 343518), CD34–APC/Cy7 (BioLegend, 343514), SLAMF6–PE (BioLegend, 317208), PE-conjugated mIgG1 isotype control (BioLegend, 400114), CD200–PE/Cy7 (BioLegend, 399806), CD47–AF647 (BioLegend, 127510), CD84–APC (BioLegend, 326009), CD274–APC/R700 (BD Biosciences, 565188), CD244–AF700 (BioLegend, 329525), CD45–APC/H7 (BD Biosciences, 641417), CD279–BV421 (BioLegend, 329920), TIGIT–BV421 (BioLegend, 372709), CD127–BV421 (BioLegend, 351309), CD45RA–BV510 (BioLegend, 740186), CD4–BV711 (BD Biosciences, 563028), LAG3–BV785 (BioLegend, 369321), CTLA4–BV785 (BioLegend, 369623), CD45–FITC (BioLegend, 304006), CD25–PE (BioLegend, 302606), CCR7–PE/Cy7 (BioLegend, 567314), CD357–PE/Cy7 (BioLegend, 371224), TIM3–APC (BioLegend, 345011), CD45RO–APC (BD Biosciences, 560899), SLAMF6–AF647 (BD Biosciences, 566093) and CD8–APC-R700 (BD Biosciences, 566857). Within the CD4^+^ and CD8^+^ T cell populations, naive cells were defined as CCR7^+^CD45RA^+^, TEMRA cells were defined as CCR7^−^CD45RA^+^, effector memory cells were defined as CCR7^−^CD45RA^−^ and central memory cells were defined as CCR7^+^CD45RA^−^.

### Analysis of in-house scRNA-seq data

Raw sequencing data were demultiplexed and converted to FASTQ format using bcl2fastq software (Illumina). Single-cell read count matrices were generated using the Cell Ranger (version 3.1.0) count pipeline (10X Genomics), which aligned, filtered and quantified barcodes against the hg19/GRCh37 reference genome. Further downstream analysis was performed using Seurat (version 4.0.0)^68^. Two datasets were generated by merging individual samples: one consisting exclusively of NBM samples and another combining AML and NBM samples. Low-quality cells with fewer than 200 detected genes and apoptotic cells with ≥15% of transcripts derived from mitochondrial genes were excluded. Data normalization and variance stabilization were performed using sctransform (version 0.3.2) on the 2,000 most highly variable genes, with mitochondrial gene expression regressed out^69^. Dimensionality reduction was performed using principal component analysis (PCA), retaining 80 principal components for further analyses. Cells from NBM samples were annotated using an external multimodal single-cell dataset comprising 30,672 NBM cells analyzed alongside 25 antibodies^70^. Annotations were transferred to the local dataset using Seurat’s TransferData function^49,70^. The annotated local NBM dataset was then used as a basis to project the PCA structure onto the larger cohort, which included all samples. Average gene expression levels for all cell types within each sample were calculated using Seurat’s AverageExpression function. SLAMF6 classification for each sample was determined on the basis of prior fluorescence-activated cell sorting (FACS) analysis. T cells were analyzed separately. After removing all other cell types from the dataset, a new PCA was performed using 30 principal components, followed by dimensionality reduction with uniform manifold approximation and projection (UMAP)^71^. This T cell dataset was reclassified using the same procedure described above and cells not reclassified as T cells were excluded. Gene signatures for T cell states based on previous studies^14,29,46,47^ were computed for each T cell using Seurat’s AddModuleScore function. *SLAMF6* expression was also analyzed in an in-house scRNA-seq dataset with mononuclear cells from five NBM donors and CD34^+^ cells enriched by magnetic separation from three of the same donors^45^.

### Analysis of *SLAMF6* expression in external datasets

*SLAMF6* expression was characterized in the publicly available scRNA-seq dataset GSE185381, containing data on mononuclear cells from ten healthy donors^29^. This dataset was processed using the same procedure as the in-house dataset, with minor exceptions. Specifically, Seurat (version 5.1.0) was used instead of Seurat (version 4.0.0) and the exclusion of low-quality cells was slightly more stringent, removing cells with fewer than 500 informative genes and adding an additional parameter to exclude cells with fewer than 1,000 total molecules detected. This dataset was also normalized using log_2_ normalization instead of sctransform normalization. Annotation was performed directly using the same external dataset^70^. Average gene expression levels for all cell types within each sample were calculated using Seurat’s AggregateExpression function. Furthermore, SLAMF6 expression was analyzed in the MS dataset from the Human Proteome Map^72^ and in the following datasets from the Human Protein Atlas: ‘immune cells’ (RNA-seq)^73^, ‘single cell type’ (scRNA-seq)^74^ and ‘the Human Protein Atlas’ (RNA-seq and immunohistochemistry)^75^.

### Survival analysis in local and external datasets

Overall survival in the local Lund cohort (*n* = 37), based on SLAMF6 expression determined by flow cytometry, was visualized using survminer (version 0.4.9)^76^. The overall survival analysis in TCGA^77^ and Beat-AML^78^ cohorts was based on *SLAMF6* RNA expression. RNA-seq and survival data for 179 AML cases from the TCGA project (https://gdc.cancer.gov/about-data/publications/laml_2012) and 575 cases from the Beat-AML project (https://biodev.github.io/BeatAML2/) were analyzed. To minimize the impact of *SLAMF6* expression from lymphoid cells, the sample composition was first determined by deconvolution using LinDeconSeq^79^. Samples estimated to contain more than 15% lymphocytes were removed from further analysis, leaving 164 of 179 cases from TCGA and 455 of 575 cases from Beat-AML. Remaining samples from each dataset were divided into three groups of equal size on the basis of *SLAMF6* expression and survival was visualized using survminer (version 0.4.9).

### Knockout of *SLAMF6* by CRISPR–Cas9

*SLAMF6*-knockout cells were generated using the Alt-R CRISPR–Cas9 System (Integrated DNA Technologies), in which Cas9 protein, *SLAMF6*-specific CRISPR RNA (crRNA) and fluorochrome-conjugated *trans*-activating crRNA were electroporated into the SLAMF6-positive human AML cell lines HNT-34 and KG-1 (DSMZ), followed by FACS and expansion of *SLAMF6*-knockout cells. Verification of *SLAMF6* knockout was performed by flow cytometry (BioLegend, 317208).

### RNA-seq of *SLAMF6*-knockout cells

RNA-seq libraries for *SLAMF6*-knockout and wild-type HNT-34 cells were produced using the stranded mRNA prep kit (Illumina) and sequenced on a Novaseq 6000 system (Illumina). The transcriptome reads were aligned to human reference genome GRCh38.109 using STAR (version 2.7.9a)^80^ and gene expression was determined as transcripts per kilobase million using salmon (version 1.10.1)^81^. Variance filtering, hierarchical clustering and *t*-test between *SLAMF6*-knockout and wild-type samples were performed using Qlucore Omics Explorer (version 3.9) (Qlucore). The *t*-test results were visualized as a volcano plot using EnhancedVolcano (version 1.24.0)^82^. GSEA^83^ was performed using fgsea (version 1.31.6)^84^ and clusterprofiler (version 4.13.4)^85^ with the Reactome^86^ gene set collection from MSigDB (version 2024.1.Hs)^83^.

### Antibody generation and binding to SLAMF6

The TNC-1 antibody was generated by phage-display scFv library screening (SciLifeLab DDD Platform) with biotinylated SLAMF6 (Innovagen). Upon conversion to full-length LALA-mutant hIgG1, binding to SLAMF6 was determined by ELISA with SLAMF6-ECD (Innovagen) and by flow cytometry in which wild-type and *SLAMF6*-knockout KG-1 cells were incubated with the antibody or an isotype control at 10 μg ml^−1^, followed by incubation with a secondary anti-human antibody (Invitrogen, H10120). For competitive binding studies, primary human T cells were preincubated with 1 mg ml^−1^ SLAMF6-ECD for 20 min at 4 °C before repeated washing and incubation with 1 μg ml^−1^ FLAG-tagged scFv fragment of the TNC-1 antibody, followed by detection with a secondary anti-FLAG antibody (Sigma-Aldrich, F4049). Requests for the TNC-1 antibody can be made to Lead Biologics International.

### Epitope mapping by HDX-MS

Epitope mapping was performed by HDX-MS using a Fab fragment of the TNC-1 antibody and automated sample preparation on a LEAP H/D-X PAL platform (Trajan) interfaced to a liquid chromatography (LC)–MS system, comprising an Ultimate 3000 micro-LC coupled to an Orbitrap Q Exactive Plus MS (Thermo Fisher). PEAKS Studio X (Bioinformatics Solutions) was used for peptide identification after pepsin digestion of undeuterated samples. Peptides identified by PEAKS with a peptide score value of logP > 25 and no modifications were used to generate a peptide list containing peptide sequence, charge state and retention time used for HDX data analysis and visualization with HDExaminer (version 3.1.1; Sierra Analytics). Observed epitopes were mapped on AlphaFold model AF-Q96DU3-F1 (DeepMind).

### NY-ESO vector design

The coding sequence of a single-chain peptide–HLA-A0201 (pMHC) construct (Addgene) carrying the NY-ESO peptide 157-165 (SLLMWITQC) was cloned into a modified pHR′SIN:CSW vector containing an SFFV promoter. To follow transgene expression, a T2A self-cleaving peptide followed by mTagBFP2 reporter was attached downstream to the pMHC sequence. The transgenic NY-ESO IG4 TCR (Addgene) was cloned into a modified pHR’SIN:CSW vector containing an SFFV promoter. A T2A self-cleaving peptide followed by eGFP was attached to the TCR sequence to determine transgene expression. Cloning was performed by In-Fusion Snap Assembly (Takara Bio). Constructs are available by request.

### T cell activation and killing

Primary T cells were isolated from peripheral blood from healthy donors (Miltenyi Biotec, 130-050-101; Biolegend, 480131) and in indicated cases CD4^+^ and CD8^+^ T cells were isolated by microbead separation (Miltenyi Biotec, 130-045-101 and 130-045-201). Cocultures with T cells and wild-type or *SLAMF6*-knockout HNT-34, KG-1 or THP-1 AML cells were performed in RPMI medium with 10% FBS at a 4:1, 9:1 or 19:1 ratio. SLAMF6 antibodies and isotype-matched control antibodies (LALA-mutant hIgG1) were added to indicated concentrations directly upon seeding. After 72 h, T cell activation and killing were analyzed by flow cytometry. T cell activation was determined by surface expression of CD25 and CD69 on viable CD3^+^CD33^−^CD4^+^CD8^−^ and CD3^+^CD33^−^CD4^−^CD8^+^ cells (BioLegend, 344816, 303416, 302606, 310910, 300518 and 301044). T cell killing was determined by the number of remaining viable AML cells (CD3^−^CD33^+^) using CountBright absolute counting beads (Invitrogen, C36950; BioLegend, 303416). Viability was determined by 7-AAD (BD Biosciences). T cell subpopulations based on CD45RA and CCR7 expression (BioLegend, 304142 and 353226) and surface marker expression of PDL1, PD1, LAG3, TIM3, CTLA4 and TIGIT (BioLegend, 393606, 317308, 344648, 344714, 329919, 345021, 369211, 369605 and 372705) after coculture were analyzed concurrently. For controls without T cells, AML cells were seeded at 0.1 × 10^6^ cells per ml under identical conditions. For experiments with the NY-ESO system, T cells were stimulated with CD3/CD28 beads (Thermo Fisher, 11132D) for 24 h before lentiviral transduction with a construct encoding the NY-ESO TCR and eGFP. Then 72 h after transduction, GFP^+^ cells were sorted and cultured without CD3/CD28 stimulation for 7–14 days. HNT-34 cells were lentivirally transduced with a construct encoding the corresponding pMHC complex and the blue fluorescent protein (BFP) marker protein, before sorting and expansion of BFP^+^ cells. Cocultures with T cells with and without NY-ESO and HNT-34 cells with and without pMHC were performed as described above but with a 1:9 ratio of T cells to AML cells.

### scRNA-seq

scRNA-seq was performed after coculture of HNT-34 AML cells and primary T cells with TNC-1 or an isotype-matched control antibody, as described above. After 72 h of coculture, cells were stained with 7-AAD (BD Biosciences) and the following antibodies for FACS and cite-seq analysis: CD3–PE/Cy7 (BioLegend, 344816), CD33–BV421 (BioLegend, 303416), CTLA4–totalseq-C (BioLegend, 369621), LAG3–totalseq-C (BioLegend, 369335), PD1–totalseq-C (BioLegend, 329963), TIGIT–totalseq-C (BioLegend, 372729) and TIM3–totalseq-C (BioLegend, 345049). Viable T cells and AML cells were sorted on an FACSAria Fusion (BD Biosciences) and libraries were prepared with Chromium GEM-X single-cell 5ʹ reagent kits v3 (10X Genomics) before sequencing on a Novaseq 6000 system (Illumina). Single-cell gene count matrices were determined using Cell Ranger (version 8.0.1; 10X Genomics) with reference genome GRCh38-2024-A. Further analyses were performed using Seurat (version 4.2.0). In brief, cells with fewer than 200 detected genes or more than 10% of transcripts having mitochondrial origin were excluded. Data normalization and variance stabilization were performed using sctransform (version 0.4.1) on the 3,000 most highly variable genes, with mitochondrial gene expression regressed out. Cell type prediction was performed with Seurat’s MapQuery function using a well-characterized NBM dataset as the ref.^49^. A distinct cluster containing cells predicted to be myeloid cells was assumed to contain HNT-34 cells. The remaining cells were predicted to constitute T cell and NK cell subsets. Differentially expressed genes between HNT-34 cells and the T cell subsets of each donor treated with TNC-1 and isotype were determined by Wilcoxon rank-sum test using Seurat’s FindMarker function. The differentially expressed genes between HNT-34 cells treated with TNC-1 and isotype were visualized in a volcano plot using EnhancedVolcano (version 1.24.0)^82^. GSEA was performed using fgsea (version 1.31.6)^84^ and clusterprofiler (version 4.13.4)^85^ with the Reactome^86^ and the Gene Ontology biological process^87^ gene set collections from MSigDB (version 2024.1.Hs)^83^.

### Antibody treatment of AML cells in vivo

Treatment experiments with AML cells were performed in 9–11-week-old male mice of the NOD.Cg-*Prkdc*^*scid*^*Il2rg*^*tm1Wjl*^/SzJ-SGM3 (NSG-S) mouse strain, a substrain of the NSG mouse overexpressing hGM-CSF, hIL-3 and hSCF (Jackson laboratory). Mice were sublethally irradiated (200 cGy) and transplanted with 5 × 10^6^ wild-type or *SLAMF6*-knockout HNT-34 cells by tail-vein injection. After 21 and 28 days, mice were transplanted with 1 × 10^6^ PBMCs from healthy donors, which were isolated by Lymphoprep separation (GE Healthcare). Antibody treatment with either TNC-1 (*n* = 7) or an isotype control antibody (*n* = 6) was administered by intraperitoneal injection 1 h and 72 h after each PBMC transplantation, at a dose of 2.0 mg kg^−1^ body weight. After 14 days of treatment, mice were killed and the isolated bone marrow and spleen cells were stained with a panel of 7-AAD (BD Biosciences) and the following antibodies: CD3–PE/Cy7 (BioLegend, 344816), CD33–BV421 (BioLegend, 303416), CD34–AF488 (BioLegend, 343518), CD45–APC (BD Biosciences, 555485) and SLAMF6–PE (BioLegend, 317208). Leukemic engraftment was determined by flow cytometry and defined as the percentage of hCD45^+^CD3^−^CD33^+^CD34^+^SLAMF6^+^ cells among the viable cells in each compartment. The experiment with *SLAMF6*-knockout HNT-34 cells was performed identically in 10–17-week-old male NSG-S mice (*n* = 6 for both treatment groups) and the treatment experiment without PBMCs was otherwise identically performed in 6-week-old male NSG-S mice (*n* = 5 for TNC-1 and *n* = 4 for the isotype control). All in vivo experiments were sex-matched, with sex selection based solely on animal availability as the applicability of the results is independent of sex. Mice were randomly assigned across ages to the TNC-1 or the isotype control treatment arms. Data collection and analysis were not performed blind to the conditions of the experiments. No animals or data points were excluded from the experiment.

### Antibody treatment of healthy HSPCs in vivo

Treatment experiments with CD34^+^ cells from healthy donors were performed in 8–12-week-old female mice of the NSG mouse strain (Jackson Laboratory) transplanted with CD34^+^ cord blood cells isolated by Lymphoprep separation (GE Healthcare) and subsequent CD34 positive selection using magnetic beads (Miltenyi Biotec). Mice were sublethally irradiated (200 cGy) and transplanted pairwise with CD34^+^ cord blood cells from five different donors. Treatment with TNC-1 (*n* = 5) or isotype control antibody (*n* = 5) was started when >10% human engraftment was detected in peripheral blood. Antibody treatment was administered by intraperitoneal injection twice weekly for 2 weeks at a dose of 2.0 mg kg^−1^ body weight. After 14 days of treatment, mice were killed and isolated bone marrow cells were stained with a panel of 7-AAD (BD Biosciences) and the following antibodies: CD3–APC/Cy7 (BioLegend, 300318), CD19–APC/Cy7 (BioLegend, 302218), CD33–BV421 (BioLegend, 303416), CD34–AF488 (BioLegend, 343518), CD38–BV711 (BD Biosciences, 563965) and CD45–APC (BD Biosciences, 555485). Human engraftment was determined by flow cytometry and the CD34^+^ and CD34^+^CD38^low^ cell populations were defined as the percentages of hCD45^+^CD3^−^CD19^−^CD33^−^CD34^+^CD38^+/low^ cells among the viable cells. Randomization, data collection and analysis were performed as for the AML treatment experiments. No animals or data points were excluded from the experiment.

### Statistics and reproducibility

Sample sizes were not predetermined by statistical methods but chosen on the basis of sample availability. Statistical tests were performed using Prism 10 (GraphPad Software). The Student’s *t*-test was used for two-group comparisons with normally distributed data. The Wilcoxon signed-rank test and the Mann–Whitney *U*-test were used for two-group comparisons of paired and unpaired non-normally distributed data, respectively. The Kruskal–Wallis test with Dunn’s post hoc test was used for multigroup comparisons with non-normally distributed data. All data met the assumptions of the statistical tests used. Research animals were randomly assigned to treatment arms across ages. Data collection and analysis were not performed blind to the conditions of the experiments. No data points were excluded from any experiments.

### Reporting summary

Further information on research design is available in the Nature Portfolio Reporting Summary linked to this article.

## Supplementary information

Supplementary Information Supplementary Fig. 1.

Reporting Summary

Supplementary Table 1 Supplementary Tables 1–4.

## Source data

Source Data All Figures Statistical source data.

## Data Availability

All data supporting the findings of this study are available within the paper and its Supplementary Information, with the following exceptions. RNA-seq data from the cell line HNT-34 with and without knockout of *SLAMF6* are available as FASTQ files from the European Nucleotide Archive under accession number PRJEB90909. The scRNA-seq data from cocultures with primary T cells and HNT-34 cells are available as FASTQ files from the European Genome-Phenome Archive (EGA) under accession number EGAD50000001573. The scRNA-seq data from primary AML samples are available as FASTQ files from the EGA under accession number EGAD50000001577. Access to the EGA datasets is currently available and can be requested by submitting an application to the Data Access Committee (EGAC50000000619), which is handled by the Research Data Office at Lund University (request@researchdata.lu.se). All requests from investigators seeking to use the data to examine scientific questions in line with Swedish laws and regulations are approved and data released according to the terms outlined in the data access agreements. In addition, processed data are available as count matrices from the SciLifeLab figshare data repository (10.17044/scilifelab.28033754 for RNA-seq from HNT-34 with and without knockout of *SLAMF6*; 10.17044/scilifelab.28033793 for scRNA-seq data from cocultures with primary T cells and HNT-34 cells; 10.17044/scilifelab.28263911 for scRNA-seq data from primary AML samples). External scRNA-seq data for validating SLAMF6 expression in NBM were downloaded from the Gene Expression Omnibus under accession number GSE185381. External RNA-seq and survival data were accessed online (https://gdc.cancer.gov/about-data/publications/laml_2012 for TCGA AML dataset; https://biodev.github.io/BeatAML2 for Beat-AML dataset). Source data are provided with this paper.

## Code availability

No new code was developed in this study.
