# Supplementary material for: Aberrant expression of SLAMF6 constitutes a targetable immune escape mechanism in acute myeloid leukemia
Source: Nat Cancer. 2025 Oct 3;6(11):1821–38. doi: 10.1038/s43018-025-01054-6 (PMC12643940; doi:10.1038/s43018-025-01054-6)
Supplement: Supplementary file 1 — Supplementary Fig. 1. [file 43018_2025_1054_MOESM1_ESM.pdf]

nature cancer

Supplementary information https://doi.org/10.1038/s43018-025-01054-6

Aberrant expression of SLAMF6 constitutes a targetable immune escape mechanism in acute myeloid leukemia

In the format provided by the authors and unedited

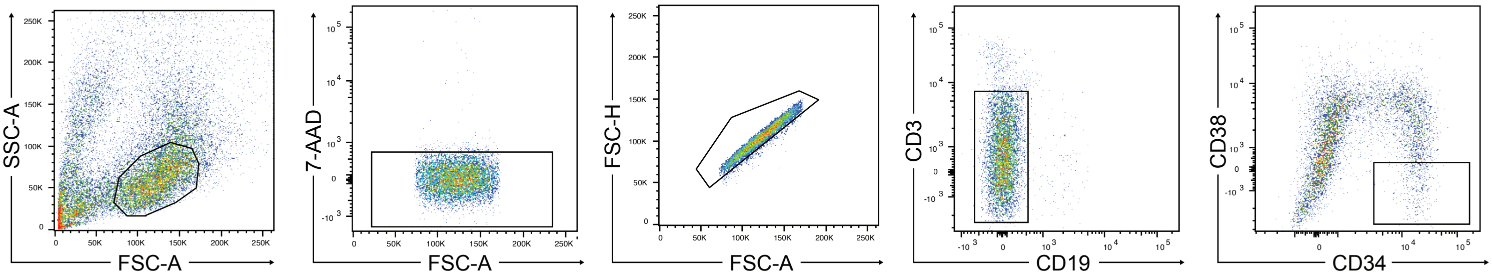

Supplementary Figure 1. Gating strategy for identification of SLAMF6 expression in antibody screen.
