# Supplementary material for: Aberrant expression of SLAMF6 constitutes a targetable immune escape mechanism in acute myeloid leukemia
Source: Nat Cancer. 2025 Oct 3;6(11):1821–38. doi: 10.1038/s43018-025-01054-6 (PMC12643940; doi:10.1038/s43018-025-01054-6)
Supplement: Supplementary file 2 — Reporting Summary [file 43018_2025_1054_MOESM2_ESM.pdf]

nature portfolio

Corresponding author(s): Carl Sandén
Thoas Fioretos

Last updated by author(s): Sep 17, 2025

Reporting Summary

Nature Portfolio wishes to improve the reproducibility of the work that we publish. This form provides structure for consistency and transparency in reporting. For further information on Nature Portfolio policies, see our Editorial Policies and the Editorial Policy Checklist.

Statistics

For all statistical analyses, confirm that the following items are present in the figure legend, table legend, main text, or Methods section.

n/a	Confirmed	
☐	☒	The exact sample size (*n*) for each experimental group/condition, given as a discrete number and unit of measurement
☐	☒	A statement on whether measurements were taken from distinct samples or whether the same sample was measured repeatedly
☐	☒	The statistical test(s) used AND whether they are one- or two-sided<br>*Only common tests should be described solely by name; describe more complex techniques in the Methods section.*
☒	☐	A description of all covariates tested
☐	☒	A description of any assumptions or corrections, such as tests of normality and adjustment for multiple comparisons
☐	☒	A full description of the statistical parameters including central tendency (e.g. means) or other basic estimates (e.g. regression coefficient) AND variation (e.g. standard deviation) or associated estimates of uncertainty (e.g. confidence intervals)
☐	☒	For null hypothesis testing, the test statistic (e.g. *F*, *t*, *r*) with confidence intervals, effect sizes, degrees of freedom and *P* value noted<br>*Give P values as exact values whenever suitable.*
☒	☐	For Bayesian analysis, information on the choice of priors and Markov chain Monte Carlo settings
☒	☐	For hierarchical and complex designs, identification of the appropriate level for tests and full reporting of outcomes
☒	☐	Estimates of effect sizes (e.g. Cohen's *d*, Pearson's *r*), indicating how they were calculated

Our web collection on statistics for biologists contains articles on many of the points above.

Software and code

Policy information about availability of computer code

Data collection	FACSDiva 8, PEAKS Studio X
Data analysis	FlowJo 10, AlphaFold, HDExaminer 3.1.1, GraphPad Prism 10

For manuscripts utilizing custom algorithms or software that are central to the research but not yet described in published literature, software must be made available to editors and reviewers. We strongly encourage code deposition in a community repository (e.g. GitHub). See the Nature Portfolio guidelines for submitting code & software for further information.

Data

Policy information about availability of data

All manuscripts must include a data availability statement. This statement should provide the following information, where applicable:

- Accession codes, unique identifiers, or web links for publicly available datasets
- A description of any restrictions on data availability
- For clinical datasets or third party data, please ensure that the statement adheres to our policy

All data supporting the findings of this study are available within the paper and its supplementary information, with the following exceptions. RNA-seq data from the cell line HNT-34 with and without knockout of SLAMF6 is available as fastq files from the European Nucleotide Archive (ENA) at accession number PRJEB90909 (https://www.ebi.ac.uk/ena/browser/view/PRJEB90909). The scRNA-seq data from co-cultures with primary T cells and HNT-34 cells is available as fastq files from the European Genome-Phenome Archive (EGA) at accession number EGAD50000001573 (https://ega-archive.org/datasets/EGAD50000001573). The scRNA-seq

data from primary AML samples is available as fastq files from the European Genome-Phenome Archive (EGA) at accession number EGAD50000001577 (https://ega-archive.org/datasets/EGAD50000001577). Access to the EGA datasets is currently available and can be requested by submitting an application to the Data Access Committee (EGAC50000000619), which is handled by the Research Data Office at Lund University (request@researchdata.lu.se). All requests from investigators seeking to use the data to examine scientific questions in line with Swedish laws and regulations are approved and data released according to the terms outlined in the data access agreements. In addition, processed data is available as count matrices from the SciLifeLab FigShare Data Repository (https://figshare.scilifelab.se/) at the following DOI:s: https://doi.org/10.17044/scilifelab.28033754 (RNA-seq from HNT-34 with and without knockout of SLAMF6), https://doi.org/10.17044/scilifelab.28033793 (scRNA-seq data from co-cultures with primary T cells and HNT-34 cells), and https://doi.org/10.17044/scilifelab.28263911 (scRNA-seq data from primary AML samples). External scRNA-seq data for validating SLAMF6 expression in normal bone marrow was downloaded from Gene Expression Omnibus, accession GSE185381. External RNA-seq and survival data were downloaded from https://gdc.cancer.gov/about-data/publications/laml_2012 (TCGA AML dataset) and https://biodev.github.io/BeatAML2 (Beat-AML dataset).

Research involving human participants, their data, or biological material

Policy information about studies with human participants or human data. See also policy information about sex, gender (identity/presentation), and sexual orientation and race, ethnicity and racism.

Reporting on sex and gender: Primary leukemia samples (n=50) were collected from both male (n=29) and female (n=21) patients (based on Swedish personal identification numbers) to allow for equitable implementation of clinically relevant findings.

Reporting on race, ethnicity, or other socially relevant groupings: The study does not contain any data on socially constructed or socially relevant categorization variables.

Population characteristics: The study population consists of AML patients from a consecutive cohort of cases, selected solely on the basis of genetic aberrations and sample availability, in order to obtain a cohort with adequate cell numbers and sufficient sample sizes for all major genetic subtypes as well as relapsed/refractory AML. No selection was performed based on disease history, treatment, age, sex/gender or other variables. The patient characteristics are detailed in Supplementary Table S1.

Recruitment: Samples were selected from a cohort of consecutive AML patients diagnosed at the Skåne University Hospital in Lund, based solely on genetic classification and sample availability. Patient inclusion was dependent on informed consent, which could bias the cohort towards patients amenable to research perticipation. However, this is unlikely to correlate with the somatic genetic aberrations studied in this project and impact the results of the study. Healthy bone marrow donors received modest financial compensation. No other compensation was awarded for study participation.

Ethics oversight: The research complies with all relevant ethical regulations. Primary samples were collected at Skåne university hospital after written informed consent from patients and in accordance with the Declaration of Helsinki. Experiments with primary leukemia samples were approved by the Swedish Ethical Review Authority (Dnr 2023-01550). Aspects of the study involving research animals were conducted in accordance with local ethical regulations and approved by the regional Animal Ethics Committee of Malmö/Lund (#7071/2020).

Note that full information on the approval of the study protocol must also be provided in the manuscript.

Field-specific reporting

Please select the one below that is the best fit for your research. If you are not sure, read the appropriate sections before making your selection.

☒ Life sciences ☐ Behavioural & social sciences ☐ Ecological, evolutionary & environmental sciences

For a reference copy of the document with all sections, see nature.com/documents/nr-reporting-summary-flat.pdf

Life sciences study design

All studies must disclose on these points even when the disclosure is negative.

Sample size: The sample size of the patient cohort was determined based on sample availability.

Data exclusions: No data exclusion.

Replication: To ensure reproducability, experiments were repeated as described in the manuscript.

Randomization: Mice were randomized into one treatment arm and one control arm.

Blinding: Blinding was not performed for in vivo treatment experiments since all samples underwent identical treatment and data from all samples underwent identical processing.

Reporting for specific materials, systems and methods

We require information from authors about some types of materials, experimental systems and methods used in many studies. Here, indicate whether each material, system or method listed is relevant to your study. If you are not sure if a list item applies to your research, read the appropriate section before selecting a response.

Materials & experimental systems

n/a	Involved in the study
☐	☒ Antibodies
☐	☒ Eukaryotic cell lines
☒	☐ Palaeontology and archaeology
☐	☒ Animals and other organisms
☒	☐ Clinical data
☒	☐ Dual use research of concern
☒	☐ Plants

Methods

n/a	Involved in the study
☒	☐ ChIP-seq
☐	☒ Flow cytometry
☒	☐ MRI-based neuroimaging

Antibodies

Antibodies used

CD45-APC (BD Biosciences #555485), CD25-PE (Biolegend #302606), CD69-APC (Biolegend #310910), CD34-AF488 (Biolegend #343518), CD38-BV711 (Biolegend #303528), CD3-PE/Cy7 (Biolegend #344816), CD7-BV711 (Biolegend #564018), CD11c-BV711 (Biolegend #301630), CD14-BV421 (Biolegend #301830), CD16-APC (Biolegend #302012), CD19-APC/Cy7 (Biolegend #302218), CD56-AF488 (Biolegend #318312), CD123-AF488 (Biolegend #306036), HLA-DR-APC (Biolegend #307610), SLAMF6-PE (Biolegend #317208), PE-conjugated mIgG1 isotype control (Biolegend #400114), LEGENDScreen v. 70001 (Biolegend #700011), LEGENDScreen v. 70007 (Biolegend #700011), CD96-BV421 (Biolegend #338418), IL1RAP-BV421 (BD #748107), CD38-BV711 (Biolegend #303528), CD34-AF488 (Biolegend #343518), CD34-APC/Cy7 (Biolegend #343514), SLAMF6-PE (Biolegend #317208), PE-conjugated mIgG1 isotype control (Biolegend #400114), CD200-PE/Cy7 (Biolegend #399806), CD47-AF647 (Biolegend #127510), CD84-APC (Biolegend #326009), CD274-APC/R700 (BD #565188), CD244-AF700 (Biolegend #329525), CD45-APC/H7 (BD #641417), CD279-BV421 (Biolegend #329920), TIGIT-BV421 (Biolegend #372709), CD127-BV421 (Biolegend #351309), CD45RA-BV510 (Biolegend #740186), CD4-BV711 (BD #563028), LAG3-BV785 (Biolegend #369321), CTLA4-BV785 (Biolegend #369623), CD45-FITC (Biolegend #304006), CD25-PE (Biolegend #302606), CCR7-PE/Cy7 (Biolegend #567314), CD357-PE/Cy7 (Biolegend #371224), TIM3-APC (Biolegend #345011), CD45RO-APC (BD #560899), SLAMF6-AF647 (BD #566093), CD8-APC-R700 (BD #566857) and TNC-1 (generated in house). All antibodies were used at a 1:20 dilution, in accordance with the manufacturers' recommendations.

Validation

The specificity of the generated SLAMF6 antibody was validated by staining of SLAMF6 wildtype and knockout KG-1 cells, as detailed in the manuscript. Commercial antibodies were validated by the manufacturers.

Eukaryotic cell lines

Policy information about cell lines and Sex and Gender in Research

Cell line source(s)

The CMK, KG-1, HNT-34, K562, MonoMac6, NB4, OCI-AML3, TF-1 and THP-1 cell lines were purchased from DSMZ, Braunschweig, Germany.

Authentication

All cell lines were authenticated by genotyping, last performed in July 2024 (Eurofins Scientific, Luxembourg City, Luxembourg).

Mycoplasma contamination

Cell lines were routinely tested for mycoplasma.

Commonly misidentified lines (See ICLAC register)

None included.

Animals and other research organisms

Policy information about studies involving animals; ARRIVE guidelines recommended for reporting animal research, and Sex and Gender in Research

Laboratory animals

Mice of the NSG and NSG-S strains (Jackson Laboratory), transplanted at 6-18 weeks of age.

Wild animals

n/a

Reporting on sex

All in vivo experiments were sex-matched to eliminate sex as a confounding factor, with sex selection based solely on animal availability since the applicability of the results is independent of sex.

Field-collected samples

n/a

Ethics oversight

Aspects of the study involving research animals were conducted in accordance with local ethical regulations and approved by the regional Animal Ethics Committee of Malmö/Lund (#7071/2020).

Note that full information on the approval of the study protocol must also be provided in the manuscript.

Plants

Seed stocks

Report on the source of all seed stocks or other plant material used. If applicable, state the seed stock centre and catalogue number. If plant specimens were collected from the field, describe the collection location, date and sampling procedures.

Novel plant genotypes

Describe the methods by which all novel plant genotypes were produced. This includes those generated by transgenic approaches, gene editing, chemical/radiation-based mutagenesis and hybridization. For transgenic lines, describe the transformation method, the number of independent lines analyzed and the generation upon which experiments were performed. For gene-edited lines, describe the editor used, the endogenous sequence targeted for editing, the targeting guide RNA sequence (if applicable) and how the editor was applied.

Authentication

Describe any authentication procedures for each seed stock used or novel genotype generated. Describe any experiments used to assess the effect of a mutation and, where applicable, how potential secondary effects (e.g. second site T-DNA insertions, mosiacism, off-target gene editing) were examined.

Flow Cytometry

Plots

Confirm that:

☒ The axis labels state the marker and fluorochrome used (e.g. CD4-FITC).

☒ The axis scales are clearly visible. Include numbers along axes only for bottom left plot of group (a 'group' is an analysis of identical markers).

☒ All plots are contour plots with outliers or pseudocolor plots.

☒ A numerical value for number of cells or percentage (with statistics) is provided.

Methodology

Sample preparation

Bone marrow aspirates and peripheral blood samples were collected at Skåne university hospital after written informed consent in accordance to the Declaration of Helsinki. Samples were collected from patients with AML or myelodysplastic syndrome (MDS) and healthy controls. Mononuclear cells were isolated using Lymphoprep (GE Healthcare, Sweden) and viably frozen before thawing and staining for flow cytometry. Bone marrow mononuclear cells from TP53-mutated AML cases (n=3) and healthy normal bone marrow (NBM) donors (n=3) were stained at 4 degrees C for 20 minutes and subsequently washed and resuspended for flow cytometry on an LSR Fortessa (BD Biosciences, USA).

Instrument

BD LSR Fortessa

Software

FACSDiva 8, FlowJo 10.

Cell population abundance

No cell sorting was performed.

Gating strategy

For identification of surface marker expression in the antibody screen, cells were first gated based on FSC/SSC, followed by dead cell exclusion based on 7-AAD/FSC, doublet exclusion based on FSC-A/FSC-H, exclusion of T and B cells based on CD3/CD19 and identification of stem and progenitor cells based on CD34/CD38. An example of the gating strategy is provided in Supplementary Figure 1.

☒ Tick this box to confirm that a figure exemplifying the gating strategy is provided in the Supplementary Information.
